# msGBS: A new high‐throughput approach to quantify the relative species abundance in root samples of multispecies plant communities

**DOI:** 10.1111/1755-0998.13278

**Published:** 2020-11-12

**Authors:** Cornelis A. M. Wagemaker, Liesje Mommer, Eric J. W. Visser, Alexandra Weigelt, Thomas P. van Gurp, Maarten Postuma, Annemiek E. Smit‐Tiekstra, Hans de Kroon

**Affiliations:** ^1^ Department of Experimental Plant Ecology Institute for Water and Wetland Research Radboud University Nijmegen The Netherlands; ^2^ Plant Ecology and Nature Conservation Group Wageningen University and Research Wageningen The Netherlands; ^3^ Systematic Botany and Functional Biodiversity Faculty of Life Sciences University of Leipzig Leipzig Germany; ^4^ German Centre for Integrative Biodiversity Research (iDiv) Halle‐Jena‐Leipzig Leipzig Germany; ^5^ Naktuinbouw Roelofarendsveen The Netherlands

**Keywords:** biodiversity, DNA, genotyping by sequencing, high throughput sequencing, roots, species abundance

## Abstract

Plant interactions are as important belowground as aboveground. Belowground plant interactions are however inherently difficult to quantify, as roots of different species are difficult to disentangle. Although for a couple of decades molecular techniques have been successfully applied to quantify root abundance, root identification and quantification in multispecies plant communities remains particularly challenging. Here we present a novel methodology, multispecies genotyping by sequencing (msGBS), as a next step to tackle this challenge. First, a multispecies meta‐reference database containing thousands of gDNA clusters per species is created from GBS derived High Throughput Sequencing (HTS) reads. Second, GBS derived HTS reads from multispecies root samples are mapped to this meta‐reference which, after a filter procedure to increase the taxonomic resolution, allows the parallel quantification of multiple species. The msGBS signal of 111 mock‐mixture root samples, with up to 8 plant species per sample, was used to calculate the within‐species abundance. Optional subsequent calibration yielded the across‐species abundance. The within‐ and across‐species abundances highly correlated (*R* ^2^ range 0.72–0.94 and 0.85–0.98, respectively) to the biomass‐based species abundance. Compared to a qPCR based method which was previously used to analyse the same set of samples, msGBS provided similar results. Additional data on 11 congener species groups within 105 natural field root samples showed high taxonomic resolution of the method. msGBS is highly scalable in terms of sensitivity and species numbers within samples, which is a major advantage compared to the qPCR method and advances our tools to reveal hidden belowground interactions.

## Abstract 2

see also the Perspective by Josep Piñol

## 1 INTRODUCTION

Our understanding of root distributions is limited compared to our knowledge of the patterning of leaves and shoots. This difference is largely due to methodological challenges as roots of different species can generally not be identified visually. With the introduction of DNA‐based detection techniques (e.g. Bobowski et al., 1999; Jackson et al., 1999; Jones et al., 2011; Linder et al., 2000; Mommer et al., 2011), the first steps were taken in opening the “black box of the underground”. Until 2008 these techniques were based on classic PCR amplification of nuclear, chloroplast or mitochondrial plant barcode loci, often combined with Sanger sequencing or RFLP (e.g. Bobowski et al., 1999; Brunner et al., 2001; Jackson et al., 1999; McNickle et al., 2008; Ridgway et al., 2003; Wildová, 2004). Individual root segments were identified on the basis of obtained PCR product length, DNA sequence or RFLP pattern. In some studies the species abundances were estimated after identification of numerous single root segments isolated from a single root core (Frank et al., 2015; Kesanakurti et al., 2011).

Mommer et al. (2008) and McKay et al. (2008) were the first to introduce quantitative polymerase chain reaction (qPCR) in studies on plant root distributions. Rather than extracting DNA from individual root segments, Mommer et al. (2008) extracted DNA from multispecies root samples. In a four‐species model system, the across‐species abundance of root samples was estimated by relating the qPCR signals from root mixtures of unknown assembly to the qPCR signals of hand‐mixed root samples of equal biomass proportions (i.e. calibration samples). In addition, species‐specific primers, rather than universal primers were used. This method was later successfully applied in biodiversity experiments using plant mixtures with up to eight species (e.g. Hendriks et al., 2015; Mommer et al., 2010; Oram et al., 2018; Padilla et al., 2013; Zeng et al., 2017).

Although many successful uses, there are three main drawbacks of using qPCR all connected to the use of species‐specific primers; (a) the primer development for each new species and the increased difficulty of it if species are more related; (b) the variable sensitivity of these primers; and (c) each species has to be analysed separately. These drawbacks inspired us to explore the use of high throughput sequencing (HTS) for the quantification of relative species abundance in mixed root samples.

DNA sequence identification and counts can be used for both species‐ identification and quantification. Hiiesalu and colleagues (2012) were first to apply HTS in the field of root ecology, using the 454 Life Sciences sequencing platform. Hiiesalu et al. (2012) showed the power of HTS, but the use of a single barcoding marker resulted in insufficient taxonomic resolution; the 37 species identified aboveground were represented by 29 belowground molecular operational taxonomic units (MOTUs). Matesanz et al. (2019) sequenced a 517 bp chloroplast rbcl marker using Miseq to analyse the root proportions of five shrubland dominant species but recorded insufficient biomass versus sequence reads correlations and high false positive rates. Lang et al. (2019) used the combined sequence information of 65 to 71 chloroplast protein coding genes (PCG) within “genome skims” (low‐coverage, short‐read sequence data sets) to estimate pollen donor proportions within pollen mixtures using. However, for two out of six pollen donor species the taxonomic resolution was still insufficient. The use of genome skims to map to a small set of genes is very data inefficient, even more when applied on roots which contain much lower number of plastids (Bramham & Pyke, 2017). Peel et al. (2019) described RevMet; 49 wild reference species were represented by genome skims which were mapped to individual long Minion sequence reads derived from mixed species pollen samples. Each read was assigned to a plant species and species proportions calculated from the collection of identified reads. The method was validated using six replicate mock pollen mixtures of known composition. This elegant approach shows promise but struggled with false positive assignments within one of the two congener plant species pairs. Root and bee pollen grains have in common that they host many Fungi (Brundrett, 2004; Leidenfrost et al., 2020) which influence the taxonomic resolution and the quantification of plant species proportions. Ondov et al. (2019) introduced Mash Screen, a MinHash (Ondov et al., 2016) based approach which enables containment estimates for every NCBI RefSeq genome within every SRA metagenome. Mash Screen has not been validated for quantification of species abundances in plant mixtures but has great potential. While current tests are still limited, the results so far suggest that none of the currently available methods are able to accurately identify all species in mixed samples.

In this paper we describe the application of genotyping by sequencing (GBS) (Elshire et al., 2011) on multiple species root samples (msGBS) which, combined with a gDNA cluster filtering strategy, has the potential of increasing taxonomic resolution. GBS is developed for SNP detection; gDNA is fragmented using endonucleases and a set of two synthetic dsDNA adapters ligated to the fragments. Due to this preparation only a subset of the full genome is PCR amplified. GBS provides a middle ground between targeted‐ and whole‐genome shotgun barcoding. The sequenced subset is clustered into a relative small reference genome which, in msGBS, is enriched for species unique clusters increasing the taxonomic resolution.

The msGBS method we developed was aimed for two purposes: (a) Quantify within‐species abundance in mixed root samples in one single molecular analysis with unprecedented taxonomic resolution; and (b) link the within‐species abundance to root biomass across‐species abundance using the calibration procedure sensu Mommer et al. (2008). msGBS was succesfully applied by in 't Zandt (2020) to asses local soil legacy effects in a multispecies grassland community.

## 2 MATERIALS AND METHODS

### 2.1 Experimental setup – Jena field study

**Figure 1:**
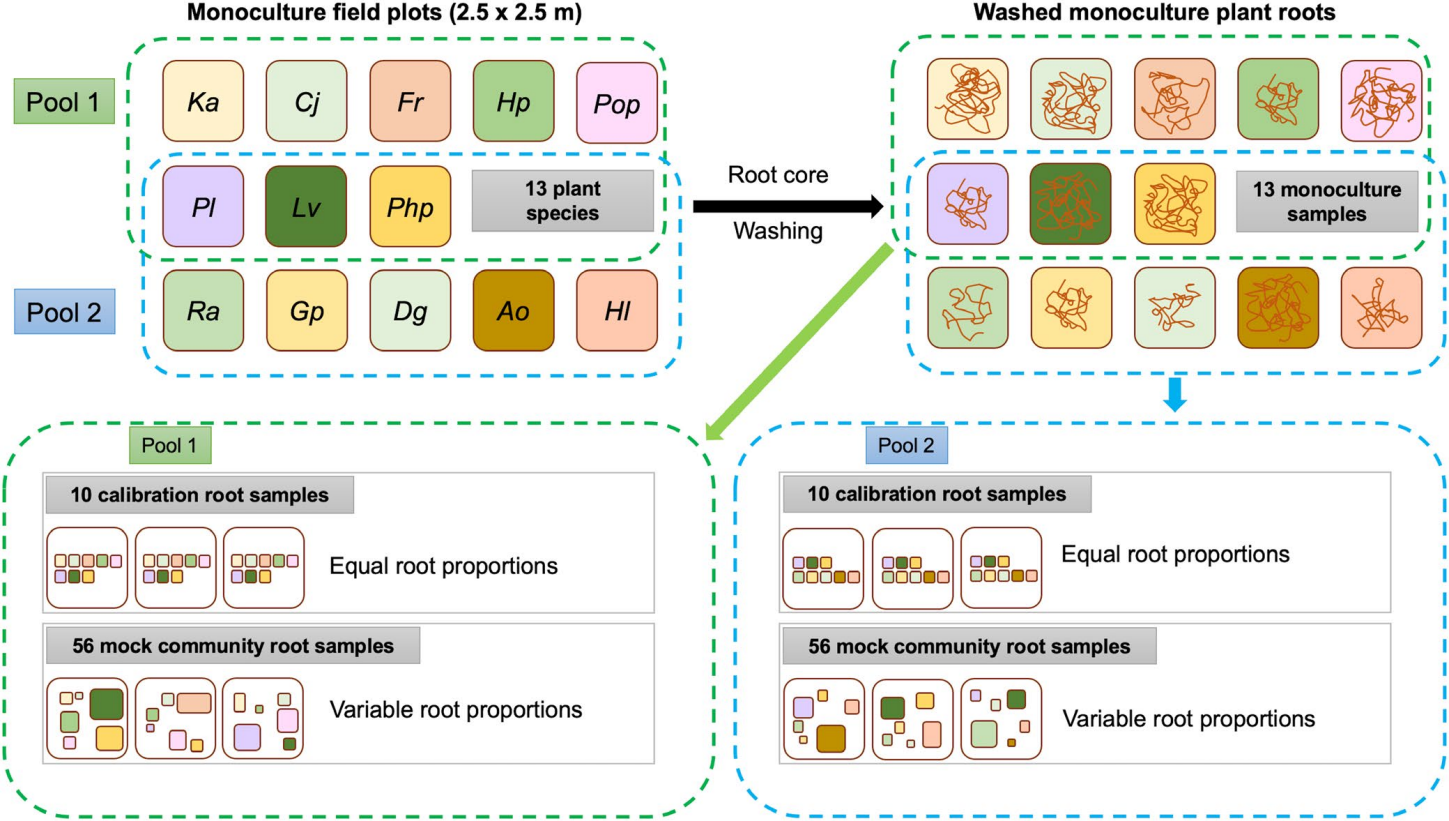
The Jena field study experimental setup. The monoculture, calibration and mock‐mixture samples are assembled from washed monoculture root material from 2.5 m × 2.5 m monoculture field plots. Two species pools were created each consisting of 8 of the 13 species; these assemblies correlated to two 8‐species field plots of the Jena experiment

The root samples used in this study were derived from the Jena Trait‐Based Experiment (Barry et al., 2019; Ebeling et al., 2014; Oram et al., 2018), with two separate species pools. Pool 1 consisted of four forbs (*Centaurea jacea* L.*, Knautia arvensis* (L.) Coult.*, Leucanthemum vulgare* Lam.*, Plantago lanceolata* L.) and four grasses (*Festuca rubra* L.*, Helictotrichon pubescens* Huds.*, Phleum pratense* L.*, Poa pratensis* L.). Pool 2 also consisted of four forbs (*Geranium pretense* L.*, Leucanthemum vulgare, Plantago lanceolata, Ranunculus acris* L.) and four grasses (*Anthoxanthum odoratum* L.*, Dactylis glomerata* L.*, Holcus lanatus* L.*, Phleum pratense*). Three species, the forbs *Leucanthemum* and *Plantago* and the grass *Phleum*, were present in both pools (Figure 1). Monoculture and mixed field plots from both pools with up to eight species were originally studied. All plots were mown twice yearly and weeded three times a year. Root cores of both pools were collected in 2016 (Oram et al., 2018) and carefully washed (debris, seeds, tubers, stolon's and taproots were carefully removed). The monoculture root material was used for the assembly of 13 “monoculture”, 20 “calibration” and 111 “mock‐mixture” samples:

- The 10 calibration samples per species pool were assembled from monoculture root material in equal per species proportions.
- 56 and 55 mock‐mixture samples of pool 1 and 2, respectively, were assembled from monoculture root material of each of eight species per pool and varied in proportions from 0% to 50%.

gDNA was extracted for these and the “unknown” mixed field plot samples and subsequently analysed by qPCR to quantify relative fine root abundances according to Mommer et al. (2008). Only the “monoculture”, “calibration” and “mock‐mixture” samples were processed using msGBS (Figure 1). The monoculture samples were processed to assemble the meta‐reference and used for downstream meta‐reference filtering, the calibration samples were used to calibrate the within‐species abundance to across‐species abundance and the mock‐mixture samples were used for the evaluation of msGBS in terms of correlations (to weighed root biomass and qPCR) and false‐positive and negative signals (FPS and FNS). Based on the FPS an analytical detection limit can be introduced (Alberdi et al., 2018; Garrido‐Sanz et al., 2020).

### 2.2 Experimental setup – Dutch field study

For the evaluation of the taxonomic resolution of msGBS we analysed the msGBS relative FPS (rFPS) of 11 congener groups within a field experiment, further referred to as the “Dutch field study”. In this field study aboveground vegetation surveys were compared to the belowground noncalibrated msGBS within‐species abundances. Leaves of in total 120 plant species (Table S1) were collected from seven field sites across a 30 km trajectory along the main branch of the river Rhine dike grasslands between the villages Ooij and Tiel in The Netherlands for meta‐reference creation. A Braun‐Blanquet (Braun‐Blanquet, 1932) vegetation survey was performed at two levels in each of the seven field sites. A 5 × 5 m^2^ plot survey and 5 1 × 1 m^2^ plots within the broader plot. From each of the 1 × 1 m^2^ plots two 40 × 400 mm root cores were taken and were subdivided in 0–10 cm, 10–20 cm and 20–40 cm depth portions, the replicate samples were combined which, after careful root washing, totalled to 105 “field mixture root” samples. gDNA was extracted from all leaf and root samples. The collected survey data and noncalibrated msGBS results was used to assess the congener (Table S1) msGBS relative false positive signals (rFPS).

### 2.3 gDNA extractions and qPCR

gDNA of the Jena field study samples were previously extracted using the DNeasy plant kit (Qiagen). The qPCR methodology and root distributions were previously described in the Jena Trait‐Based Experiment papers (Barry et al., 2019; Oram et al., 2018). gDNA of the Dutch field study samples was extracted using the Nucleospin plant II kit (MN).

### 2.4 msGBS library preparations and sequencing

The GBS protocol, as described by Elshire et al. (2011), was altered regarding the restriction enzymes and the adapter design (Figure S1 and Table S2). A more detailed lab protocol can be found in the Extended lab protocol section of the supporting information. In total three pooled sequence libraries were constructed; one for the 144 samples of the Jena field study, one for the 122 monoculture leaf samples of the Dutch field study and one for the 105 root samples of the Dutch field study. The Dutch field study samples were equimolar pooled using qPCR. Half a sequence run (lane) was used for the Jena msGBS library and a full sequence run for each of the Dutch field study msGBS libraries.

First, 300 ng of genomic DNA (gDNA) of each sample was digested by two restriction enzymes (*Pac*I and *Nsi*I) after which two indexed adapters were ligated to the DNA fragments. The main change in the adapter design was the incorporation of three random nucleotides per adapter for the identification of PCR duplicates within each amplified msGBS library. After the ligation step the samples were pooled, mixed and aliquoted in eight portions per library for practical reasons and to prevent the effect of PCR bias. For the Dutch field study the diglig reactions were equimolar pooled based on a qPCR quantification using the KAPA Library Quantification Kit for HTS (KAPA Biosystems). The aliquots were concentrated (QIAquick, Qiagen), AMPureXP size selected preferring >150 bp DNA fragments (Beckman coulter) and PCR amplified using KAPA HiFi HotStart readyMix (Roche Diagnostics). The PCR reactions were combined, QIAquick concentrated and quantified using the KAPA Library Quantification Kit for HTS. The final three pooled msGBS libraries were spiked with 10% PhiX DNA to increase the DNA complexity of the library in order to improve the Hiseq colour matrix estimation for which the first 11 sequencing cycles are used overlapping with our index region. Sequencing was performed by Novogene (Hongkong) on a Illumina (USA) Hiseq X‐Ten sequencer; 2 × 150 bp paired‐end (PE) sequencing reads, each one starting with 3 unique molecule identifiers (UMI) nucleotides and the adapter index.

### 2.5 msGBS data processing

**Figure 2:**
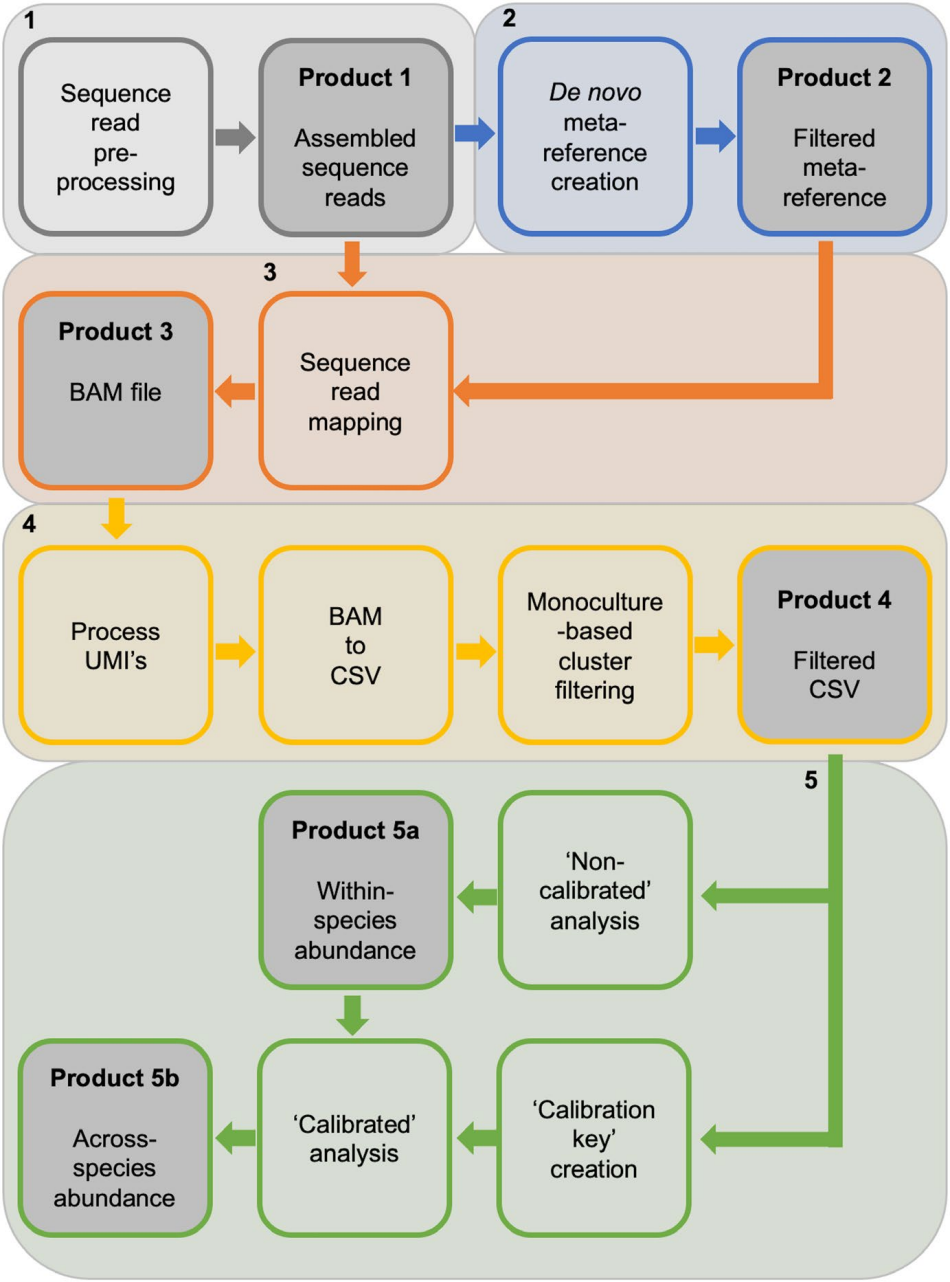
Overview of the msGBS analysis as outlined in the text and supporting documentation. Process 1 (gray) depicts the pre‐processing of the sequence reads and produces product 1; the assembled sequence reads. Process 2 (blue) is the creation of the BLASTN filtered meta‐reference (product 2). Process 3 (orange) depict the sequence read mapping to produce a BAM alignment file (product 3). Process 4 (yellow) is the identification of PCR duplicates, the conversion of BAM to CSV format and the monoculture‐based cluster filtering. Total, per sample per cluster, read counts are stored in a filtered CSV file (product 4). Process 5 (green) starts with the non‐calibrated analysis which results in the within‐species abundance (product 5a). Next, a calibration key was created from the calibration sample read counts. The calibration key was subsequently used to convert the within‐species abundance into the across‐species abundance (product 5b)

Computations were executed on a local Linux cluster node in Nijmegen, The Netherlands. Writing and debugging of Python scripts (Python Core Team, 2015) was performed using PyCharm Professional 2017 2.2. R (Suhl et al., 2014) was executed using Rstudio (RStudio Team, 2016). The msGBS data processing can be described by five processes which are outlined in Figure 2:

Process 1: Sequence read preprocessing. First, the reads were demultiplexing; the sequence read adapter indices are coupled to the sample name which was added to the read header. The 2 × 3 bp UMI nucleotides were processed and together with the indices stripped from the sequence read and added to the read header (Figure S1). Next, the reads were inspected for adapter traces and low‐quality nucleotides (<Q10) and trimmed when needed. All PE reads were merged (minimum 20 bp overlap) or else joined. The combined merged and joined reads are the assembled reads (Figure 2, product 1).

Process 2: Meta‐reference creation. For each monoculture a de novo assembled reference was created from dereplicated and clustered (with 95% identity) monoculture assembled reads. The clusters of all monoculture references are combined into a single meta‐reference (a digital gDNA sequence database) while retaining original monoculture identifier names. The meta‐reference was cleansed from all identifiable non‐Eukaryota and Fungi clusters by a local BLASTN search against the NCBI nr database (Figure S3).

Process 3: Sequence read mapping. The assembled reads from all samples were mapped to the meta‐reference. A BAM (sequence alignment file) was created (Figure 2, product 3), in which the read header information was retained.

Process 4: Post‐processing of read mapping data. First the UMI’s and the mapping alignment scores in the BAM file are processed; sequence reads are marked as “is_duplicate” or “qc_fail”, respectively. PCR duplicates are evaluated on a per meta‐reference cluster, within sample level. They can cause bias in the analysis as the duplication rate can vary between amplified regions and samples. The BAM file is converted to CSV format; only the total read counts per cluster per sample are retained, reads marked as “is_duplicate” or “qc_fail” are not counted. A minimum total read count threshold of 10 reads per cluster over all samples was set; clusters that failed this criteria were removed from the CSV file.

**Figure 3:**
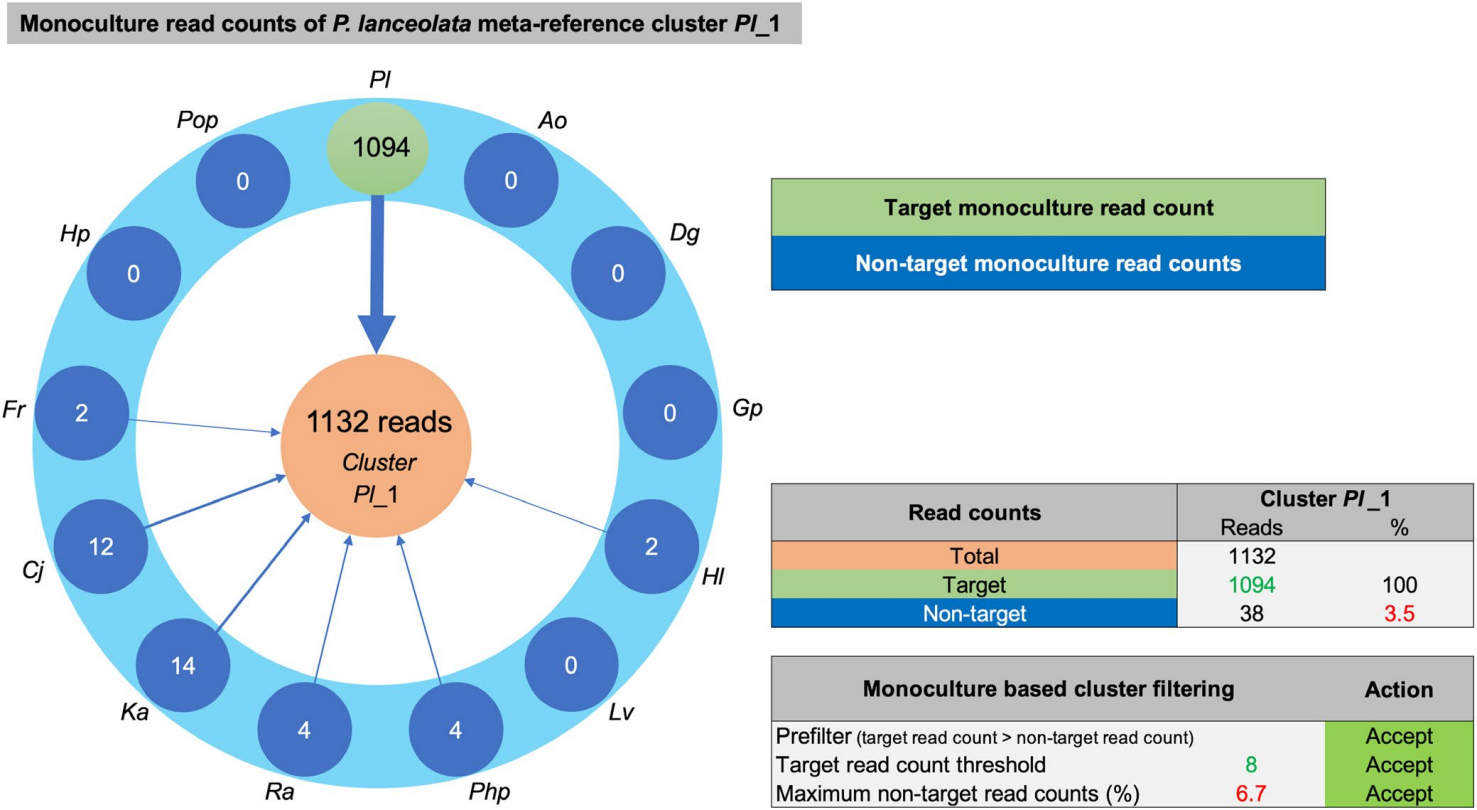
Illustration of the monoculture‐based cluster filtering (Figure 2, process 4, step 3). The monoculturebased filtering evaluates the mapped read counts of the monoculture samples. Each cluster is evaluated individually. If a read from a monoculture sample was mapped to a cluster that originated from that monoculture sample this is called a target read count and if mapped to a cluster that originated from another monoculture sample this is called a non‐target read count. In this example 1,132 reads were counted for Plantago lanceolata meta‐reference cluster Pl_1 which could be split in 1,094 target read counts and 38 (3.5%) non‐target read counts. The Pl_1 cluster did pass the all evaluation steps so this cluster was accepted and recorded in the filtered CSV file

An important step of the post‐processing is the monoculture‐based cluster filtering which uses the monoculture read counts in the CSV file to identify and discard between monoculture root sample homologous clusters. Removal of these clusters increases the taxonomic resolution of msGBS. These homologous clusters are plant‐born or nonplant‐born clusters that are present in multiple monoculture root samples. Monoculture per cluster read counts were either “target read counts” or “non‐target read counts” as illustrated in Figure 3. The read counts are evaluated by three filter steps. (a) Prefilter; questions if is the highest read count is indeed the target species. (b) Target count filter; a minimum of eight counted reads (script filter parameter f1 = 8) which enables effective nontarget filtering (step 3). (c) Non‐target count filter; the non‐target read count threshold of the Jena field study monocultures was set to 1/15th (script filter parameter f2 = 15) of the target read count. This corresponds to a maximum non‐target signal of 6.7%.

When a cluster passed all filter steps this cluster, and the reads counts of all samples, was recorded in the filtered CSV file. Finally the total number of read counts, of all filtered clusters combined, were counted for all samples. Jena field study samples for which, in total, less than 1,000 reads were counted (script filter parameter f3 = 1,000) were removed from the filtered CSV file (Figure 2, product 4).

Process 5: Noncalibrated and calibrated analysis. msGBS filtered CSV data was processed in two steps as illustrated in Table 1. The first step, which was performed for both the Jena and Dutch field study samples, is the noncalibrated analysis in which the per species read counts is divided by the total reads count of the mock‐mixture and field root mixture samples, respectively. This resulted in the within‐species abundance (Figure 2, product 5a). The second step, which was only performed for the Jena field study, is the optional calibration of the within‐species abundances. Since typical gDNA yields vary among species, we expected biomass independent, species‐specific variation in the number of reads within samples. To estimate across‐species abundance in mixed samples, the within‐species abundance thus needed to be calibrated (sensu Mommer et al., 2008). Ten calibration samples per pool, assembled from per species equal proportions of fresh monoculture root biomass, were used to calculate a calibration key. The calibration key was used to convert the within‐species abundance of the mock‐mixture samples to across‐species abundance (Figure 2, product 5b) which was subsequently projected on the total biomass.

**Table 1:** Final calculations of a mock‐mixture sample in non‐calibrated and calibrated mode. First the per species read counts of both calibration and mock‐mixture samples are divided by total read count. The relative read counts of a mock‐mixture sample is the ‘within‐species abundance’ (green) in non‐calibrated mode. For calibrated mode the averaged relative read counts of the calibration samples is called the calibration key (blue). In calibrated mode the across‐species abundance of a mock‐mixture sample is calculated in two steps. First the relative read counts of the mock‐mixture sample is divided by the calibration key which results in the calibrated msGBS signal. The across‐species abundance (red) is calculated by dividing the per species calibrated signal to the total calibrated signal

	Species of pool 1															
	*P. lanceolata*		*K. arvensis*		*C. jacea*		*F. rubra*		*H. pubescens*		*P. pratensis*		*L. vulgare*		*P. pratense*	
	Calibration samples (10)															Total
Biomass based proportions	1	:	1	:	1	:	1	:	1	:	1	:	1	:	1	
Average read counts	633	:	4,429	:	2,652	:	3,119	:	3,046	:	13,137	:	2,414	:	5,147	34,576
Calibration key	0.02	:	0.13	:	0.08	:	0.09	:	0.09	:	0.38	:	0.07	:	0.15	1

	Example mock‐mixture sample															Total
Biomass based proportions	?	:	?	:	?	:	?	:	?	:	?	:	?	:	?	
Read counts	247	:	2,259	:	1,121	:	4,291	:	14	:	6,520	:	546	:	894	15,892
Within‐species abundance	0.02	:	0.14	:	0.07	:	0.27	:	0.00	:	0.41	:	0.03	:	0.06	1
Calibrated msGBS signal	0.85	:	1.11	:	0.92	:	2.99	:	0.01	:	1.08	:	0.49	:	0.38	7.83
Across‐species abundance	0.11	:	0.14	:	0.12	:	0.38	:	0.00	:	0.14	:	0.06	:	0.05	1

Jena field study only; the FPS of the mock‐mixture samples was evaluated by calculating the averaged, per species, across‐species abundance when this species was not present in the assembly. The FNS threshold in calibrated mode was defined as 1% across‐species abundance. In noncalibrated mode the FNS threshold was defined on a per species level; we defined this threshold as 1/50th of the msGBS signal of the, for that species, 50% biomass mock‐mixture samples.

Dutch field study only; we used the within‐species abundances of 11 congener groups to calculate the average rFPS, i.e., the average msGBS (field root mixture sample) signal of absent species divided by the average msGBS signal of congener species that are present (Table S3). The actual biomass‐based proportions of these samples are unknown; samples were selected for comparison when (a) field plots were available in which not all species of a congener groups were present; and (b) when a msGBS signal for the species that was present was detected.

More details on the bioinformatics can be found in the extended bioinformatics section of the Supporting Information.

### 2.6 Statistical analysis

Regression analysis were performed for all comparisons of the biomass‐based species proportions the qPCR‐ and msGBS estimates of relative species abundance. In order to evaluate the between species variation in sequence read mapping counts of the calibration samples we calculated the coefficient of variation (CV). A two‐way ANOVA was used to test if the calibrated msGBS and qPCR results were significantly different.

## 3 RESULTS

### 3.1 msGBS library preparations and sequencing

For the Jena field study samples a total of 144 msGBS reactions were pooled into a single msGBS sequencing library (Figure S2) with a final DNA yield of 11.3 ng/μl (qPCR) and an average fragment size of 940 basepairs (bp). The sequencing yielded 217,171,278 2 × 150 bp PE msGBS reads (Table S4). For the Dutch field study samples a total of 224 msGBS reactions were equimolarly pooled in two subsets using qPCR quantification which resulted in two msGBS sequencing libraries with a final yield of 15.7 ng/μ and 3.47 ng/μ, respectively. The sequencing of these msGBS libraries resulted in 378,265,715 and 291,588,907 2 × 150 bp PE msGBS reads, respectively (Table S4).

### 3.2 Jena field study msGBS results

The results of the Jena field study msGBS data processing following Figure 2.

Process 1. Sequence preprocessing. During demultiplexing of the monoculture‐, calibration‐ and mock‐mixture samples adapter barcodes were successfully identified in 181,555,188 reads (84%). This number ranged from 67,657 to 4,505,442 per sample. Nonidentified reads originated from PhiX DNA (10.9%) or adapter dimers (6.1%). Adapter traces were identified and trimmed in 13.8% of the reads. Of the assembled reads (Figure 2, product 1) 39% and 61% of the reads were merged and joined, respectively.

Process 2. De novo meta‐reference creation. On average 102,155 meta‐reference clusters were generated per monoculture root sample; on average one cluster per 12 PE reads which results in a total of 1,328,016 clusters (Table S5). The number of clusters per monoculture varied from 8,475 to 260,885. A positive correlation (*R* ^2^ = 0.92) was found between number of processed monoculture reads and generated clusters per species (Figure S4) which implies that a higher sequencing effort will result in more clusters per species. BLASTN filtering removed 1.1% of the clusters from the meta‐reference (Table S6) which were mainly annotated as arbuscular mycorrhizal (AM) fungi and bacteria (60% and 34%, respectively).

Process 3. Sequence read mapping. In total 62% (Table S4) of the assembled reads of monoculture‐, calibration‐ and mock‐mixture samples were recorded in the BAM file. That a high proportion of reads did not map to the meta‐reference can be caused by (a) the absence of a homologous cluster due to low monoculture sequencing effort or BLASTN filtering; or (b) reads that were too short for mapping after low quality nucleotide trimming.

Process 4. On average 23.3% of the reads were marked as “is‐duplicate” and/or “quality‐fail” in the BAM file. The extracted CVS file counted 86,443,367 reads (Table S4) which were mapped to 726,605 clusters (Table S7).

The monoculture‐based cluster filtering evaluated the read counts of the 9,446,804 monoculture reads (Table S4) to the 726,605 remaining clusters in the CSV file. In total 29.4% of the clusters (213,367) were retained in the filtered CSV file (Table S7). Table 2 shows the target‐ and nontarget read counts of the monoculture samples after monoculture‐based cluster filtering. Target read counts ranged from 92.78% to 99.95%, the per species averaged nontarget read counts ranged from 0.00% to 0.60%. The combined effect of the BLASTN and monoculture‐based cluster filtering on the mock‐mixture root samples is evaluated at the end of the result section of process 5b.

**Table 2:** Evaluation of the monoculture‐based cluster filtering. The read counts are expressed relative to the monoculture sample total read count. The non‐target signals are heatmap colored

		Species pool		Total meta‐reference cluster read counts (%)													Average non‐target read counts (%)	Total read counts
		1	2	*P. lanceolata*	*R. acris*	*K. arvensis*	*G. pratense*	*C. jacea*	*D. glomerata*	*A. odoratum*	*H. lanatus*	*F. rubra*	*H. pubescens*	*P. pratensis*	*L. vulgare*	*P. pratense*		
Monoculture sample	*P. lanceolata*	X	X	98.06	0.08	0.37	0.00	0.07	0.19	0.43	0.01	0.06	0.59	0.06	0.04	0.03	0.16	23,536
	*R. acris*		X	0.18	99.65	0.05	0.00	0.01	0.00	0.01	0.02	0.00	0.00	0.05	0.01	0.02	0.03	64,911
	*K. arvensis*	X		0.14	0.02	99.60	0.00	0.02	0.09	0.01	0.01	0.01	0.00	0.07	0.02	0.02	0.03	82,920
	*G. pratense*		X	0.00	0.00	4.12	92.78	0.00	0.00	0.00	0.00	2.06	0.00	0.00	1.03	0.00	0.60	97
	*C. jacea*	X		0.19	0.04	0.13	0.00	98.52	0.02	0.00	0.09	0.19	0.05	0.49	0.09	0.19	0.12	22,645
	*D. glomerata*		X	0.00	0.01	0.05	0.00	0.05	99.24	0.10	0.00	0.18	0.11	0.13	0.08	0.05	0.06	52,738
	*A. odoratum*		X	0.01	0.03	0.01	0.00	0.04	0.01	99.58	0.06	0.13	0.02	0.01	0.08	0.02	0.04	11,862
	*H. lanatus*		X	0.00	0.00	0.00	0.00	0.00	0.00	0.00	99.95	0.01	0.01	0.01	0.00	0.00	0.00	181,146
	*F. rubra*	X		0.01	0.02	0.00	0.01	0.02	0.02	0.01	0.14	99.51	0.04	0.07	0.13	0.04	0.04	19,868
	*H. pubescens*	X		0.01	0.01	0.01	0.00	0.00	1.14	0.01	0.07	0.04	98.57	0.03	0.03	0.08	0.12	14,432
	*P. pratensis*	X		0.01	0.04	0.00	0.00	0.00	0.11	0.01	0.14	0.01	0.15	99.50	0.01	0.01	0.04	172,862
	*L. vulgare*	X	X	0.00	0.03	0.06	0.00	0.03	0.00	0.06	0.17	0.04	0.07	0.06	99.48	0.00	0.04	6,917
	*P. pratense*	X	X	0.02	0.05	0.02	0.00	0.01	0.04	0.04	0.03	0.06	0.03	0.09	0.11	99.49	0.04	116,427

**Figure 4:**
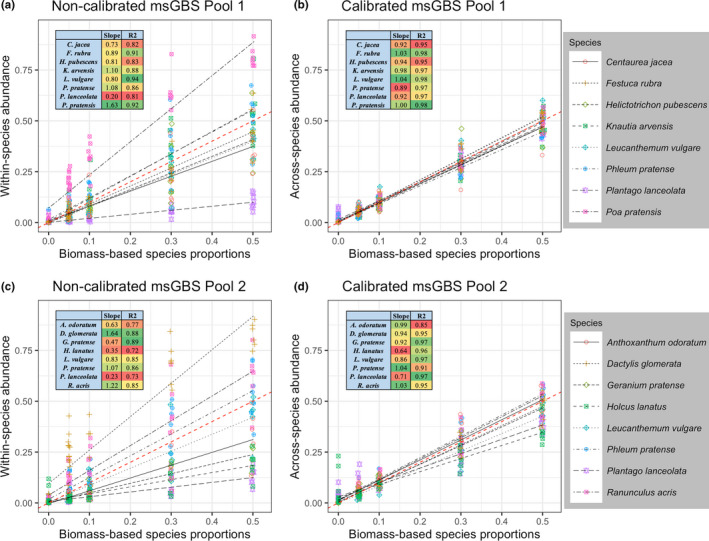
Biomass‐based species proportions compared to the msGBS in non‐calibrated and calibrated mode. Correlation of biomass‐based species proportions to the msGBS‐ non‐calibrated within‐species abundance and calibrated across‐species abundance of the mock‐mixture samples of species pool 1 (ab) and 2 (cd). Regression line slopes and correlations are inserted as a table

Process 5a. msGBS noncalibrated analysis. Figure 4a,c show that high correlations, ranging from *R* ^2^ = 0.72 to 0.94, between the within‐species abundance and the biomass‐based species proportions were found. However, the wide range of slopes (ranging from 0.20 to 1.64) show that the assessment of across‐species abundance within mixed root samples is impossible without proper calibration. The msGBS FPS in noncalibrated mode was 0.46% (Table S8). For pool 1 and 2 no msGBS FNS were found in noncalibrated mode.

Process 5b. msGBS calibrated analysis. Figure 4b,d illustrates the effect of the calibration procedure; the correction for per species typical read yield correct the slope towards 1 while high correlations, ranging from *R* ^2^ = 0.85 to 0.98, were retained. The calibration key, needed for this correction, was calculated from a set of 10 replicate calibration samples for each species pool. Indeed, there was large variation in between species read counts per unit of root biomass (Tables S9A and S10A). The averaged read counts varied from 609 to 13,298 in pool 1 and 315 to 4,597 in pool 2. This again illustrates why across‐abundances within root samples cannot be based directly on read counts. As in the qPCR method of Mommer et al. (2008), the calibration procedure is sensitive for signal variation between the calibration samples, due to errors in weighing equal tiny fresh biomasses by hand. Specifically, msGBS also requires comparable relative read counts between the calibration samples (Table S9B and S10B). Outlier values, containing one or more values that deviated more than 2.5 STD, were detected (sample 1 of pool 1 and sample 15 and 18 of pool 2) and removed (Tables S9B and S10B, Figures S5 and S6). Removal of outlier “calibration” samples is standard procedure in the old qPCR method and justified because of the sensitive root weighing procedure.

**Figure 5:**
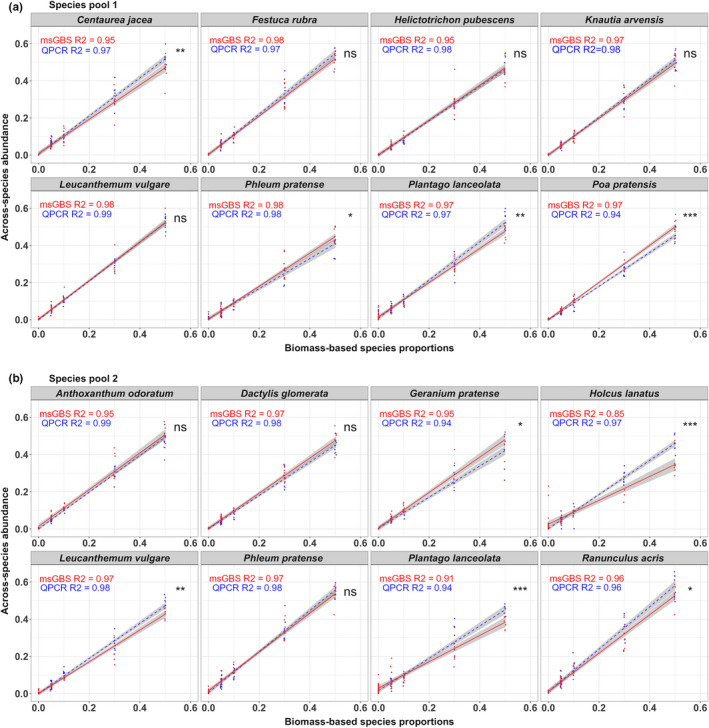
msGBS compared to qPCR. The biomass‐based species proportions are compared to the msGBS (red, solid lines) and qPCR (blue, dashed lines) across‐species abundance of the mock‐mixture samples of species pool 1 (a) 2 (b). The gray areas display the 0.95 confidence intervals. Asterisks note if msGBS and qPCR regression models are significantly different (<0.001(***), 0.001–0.01(**) and 0.01–0.1(*))

The biomass‐based species proportions of the pool 1 and 2 mock‐mixture samples was compared to the msGBS‐ and qPCR across‐species abundances (sensu Mommer et al., 2008) (Figure 5a,b). In general, we found comparable correlations ranging from *R* ^2^ = 0.84 to 0.97 for msGBS and *R* ^2^ = 0.94 to 0.98 for qPCR (Table S11 and S12). These high correlations show that msGBS in calibrated mode can reliably estimate the across‐species abundance within root mixtures. The slopes of the regression models ranged from 0.64 to 1.04 for msGBS and from 0.81 to 1.14 for qPCR. For eight out of 16 species the msGBS slopes were within the 0.95 confidence interval boundaries of the slope = 1. The regression models can be used to estimate across‐species abundance from the msGBS signal of unknown experimental samples. Further analysis indicated that msGBS average FPS (0.88%) of the mock samples are comparable to those obtained using qPCR (0.43%) (Table S13 and S14). In pool 2 *Plantago lanceolata* and *Holcus lanatus* had a relative high average msGBS FPS (4.6% and 3.5%, respectively). For pool 1 and 2 no msGBS FNS were found in calibrated mode. The msGBS FPS in calibrated mode were comparable to those in noncalibrated mode (0.42% and 0.46%, respectively, Table S8).

### 3.3 The effect of cluster filtering

The combined effect of the BLASTN‐ and monoculture‐based cluster filtering on the msGBS results was evaluated by comparing the msGBS and qPCR across‐species abundances of the mock‐mixture samples of pool 1, with‐ and without the combined filtering steps (Table S15). The average correlation (*R* ^2^) of the across‐species abundance and biomass‐based species proportions was improved from 0.95 to 0.98 by the combined optimizations. The average FPS was lowered from 2.05% to 0.42% by the combined optimizations. This demonstrates the effectivity of the BLASTN‐ and monoculture‐based filtering in quenching false positive signals.

### 3.4 The influence of species assembly on the calibration key

To assess the influence of species assembly on the calibration key values and the regression model slopes of the biomass‐based species proportions and noncalibrated msGBS estimated within‐species abundance we compared these values of the three species present in both pools (Figure S7). Due to the species assembly the slope shift ranged from 0.01 to 0.03, the calibration key shift ranged from 0.01 to 0.04 (Table S16).

### 3.5 Dutch field study msGBS results

On average 44,044 meta‐reference clusters were generated per monoculture leaf sample; on average one cluster per 76 PE reads, more reads compared to the one cluster per 12 PE reads of the Jena study monocultures. This can be explained by two reasons: (a) the Vsearch minuniquesize parameter which was increased from 2 to 3 because of the higher per monoculture sequence read input; and (b) the fact that the Jena study monocultures derived from root, rather than leaf material which is expected to contain more Bacteria and Fungi. The second reason is hypothesized to be the cause of the difference in the percentage of leaf monoculture‐ and root field sample assembled reads retained in the filtered CSV file; 18% and 6%, respectively (Table S4).

The noncalibrated msGBS data of the Dutch field study was used to evaluate the congener specificity; does the monoculture based cluster filtering effectively identify between congener species homologous clusters and sufficiently increase the taxonomic resolution? To answer this question we looked at the msGBS rFPS of ‘absent’ species (based on extensive field surveys), which is assumed to be caused by the presence of congener species. This rFPS is calculated by dividing the msGBS signal of the “absent” congener specie(s) by the msGBS signal of the “present” congener specie(s). The analysis was based on five congener pairs, five congener triplets and one congener quartet. For congener triplets and quartets comparisons were performed in all available combinations (Table 3). For five congener combinations we found less than 0.5% rFPS and for three congener combinations between 0.5% and 3% rFPS. For the remaining three congener combinations we found a higher rFPS ranging from 14.53% to 43.96%. A closer look at the actual field survey data and their msGBS within‐species abundances was used to discus these high rFPS signals (Figures S8 and S9).

**Table 3:** The performance of msGBS and RevMet in terms of rFPS within congener species groups. The average signal for RevMet is the bee pollen species abundance (%); and for msGBS, it is the non‐calibrated within‐species root abundance of the *Dutch field study* root samples. N is the number of samples included in the comparison

	Congener group	Species present in mock sample	N	Average signal (%)	Species absent in mock sample	N	Average signal (%)	rFPS (%)
RevMet	*Papaver*	*Papaver somniferum*	6	4.07	*Papaver rhoeas*	6	0.07	1.64
	*Ranunculus*	*Ranunculus repens*	12	13.94	*Ranunculus acris*	12	4.97	35.62

	Congener group	Species present aboveground	N	Average signal	Species absent aboveground	N	Average signal	rFPS (%)
msGBS	*Centaurea*	*Centaurea jacea*	30	0.051220	*Centaurea scabiosa*	30	0.000049	0.10
	*Cerastium*	*Cerastium fontanum*	20	0.000100	*Cerastium arvense*	20	0.000005	2.91
		*Cerastium glomeratum*	4	0.000528				
	*Cirsium*	*Cirsium vulgare*	18	0.005523	*Cirsium arvense*	18	0.000802	14.53^a^
	*Convolvulus*	*Convolvulus arvensis*	53	0.045996	*Convolvulus sepium*	53	0.000017	0.04
	*Euphorbia*	*Euphorbia esula*	8	0.000841	*Euphorbia helioscopia*	8	0.000003	0.41
	*Galium*	*Galium mollugo*	58	0.050836	*Galium aparine*	58	0.000037	0.07
	*Geranium*	*Geranium dissectum*	20	0.003020	*Geranium molle*	20	0.000007	0.24
					*Geranium pratense*	20	0.000025	0.84
	*Ranunculus*	*Ranunculus acris*	11	0.000336^b^	*Ranunculus repens*	22	0.000144	43.96^c^
		*Ranunculus bulbosus*	18	0.000541^b^				
	*Rumex*	*Rumex acetosa*	9	0.000309	*Rumex crispus*	9	0.000020	6.55
					*Rumex thyrsiflorus*	9	0.000096	31.12^d^
		*Rumex acetosa*	7	0.000710	*Rumex crispus*	8	0.000001	0.04
		*Rumex thyrsiflorus*	6	0.003044				
		*Rumex acetosa*	13	0.001469	*Rumex thyrsiflorus*	16	0.000348	15.99^d^
		*Rumex crispus*	9	0.003206				
	*Trifolium*	*Trifolium pratense*	15	0.004809	*Trifolium dubium*	15	0.000000	0.00
					*Trifolium repens*	15	0.000001	0.01
	*Vicia*	*Vicia cracca*	0	0.000000	*Vicia hirsuta*	24	0.000527	2.24
		*Vicia sativa*	24	0.023538	*Vicia sepium*	24	0.000007	0.03
		*Vicia cracca*	1	0.000019	*Vicia sepium*	15	0.000012	0.01
		*Vicia sativa*	14	0.053774				
		*Vicia hirsuta*	14	0.187578				

^a^ High rFPS was caused by an isolated very high signal (within 1 of the 18 samples) suggesting that the ‘absent’ congener species *Cirsium arvense* was missed in the survey of this field plot or a seed accidently remained after washing and cleanup of this root sample.

^b^ No interference between *R. acris* and *R. bulbosus*.

^c^ High rFPS caused by the presence of *R. bulbosus*.

^d^ Interference between *R. acetosa* and *R. thyrsiflorus*.

## 4 DISCUSSION

As one of the very few molecular techniques to quantify relative species abundance in mixed root samples, the qPCR method of Mommer et al. (2008) produces robust results but also has its limitations. Here we present a new molecular method that solves these drawbacks by: (a) allowing analysis of, essentially, an unlimited number of species in a single root sample; (b) the increased sensitivity to low species abundances compared to qPCR due to scalable sequencing effort; (c) the labour friendliness; (d) the prevention of PCR bias due to the use of Unique Molecule Identifiers (UMI’s); and (e) the relative low laboratory costs (32 euro per sample). Our analysis show that msGBS is a very robust high‐throughput molecular method to quantify across‐species abundance related to root biomass (in calibrated mode) or within‐species abundance across samples (in noncalibrated mode) in mixed root samples. Results of msGBS and qPCR were highly correlated in calibrated mode. msGBS had no false negative signals (FNS) and low (relative) false positive signals ([r]FPS) in most cases showing unprecedented taxonomic resolution. Out of 11 congener comparisons, only between two very closely related congener pairs significant rFPS was reported. The msGBS labwork is slightly more technical but more affordable compared to the current state of the art (RevMet).

msGBS thus outperforms other techniques on taxonomic resolution although extensive tests are yet to be performed and improvements with other techniques are possible. The taxonomic resolution, at congener level, is insufficiently validated for the currently available DNA based techniques (qPCR [Mommer et al., 2008], metabarcoding [Matesanz et al., 2019] and shotgun metabarcoding [Lang et al., 2019]). For RevMet (Peel et al., 2019) only limited congener data is available but so far high rFPS are reported for one of two tested congener pairs. Smart application of filtering strategies or MinHash (Ondov et al., 2019) based analysis might, in the near future, further increase the taxonomic resolution of HTS based sequence data and the independent of origin of the used sequences (e.g. GBS, genome skimming or MinION), especially when more NCBI RefSeq genomes become available.

### 4.1 Methodological considerations regarding msGBS

#### 4.1.1 msGBS library preparation

msGBS libraries were prepared for all 120 species of both Jena and Dutch field Study. The observed variation in number of demultiplexed sequence reads between samples is not uncommon for GBS based techniques (Gardner et al., 2014; Sonah et al., 2013), and is suggested to be the result of variation in gDNA quality (especially the presence of secondary metabolites and ethanol residues). qPCR based msGBS library pooling, as performed for Dutch field Study samples, accommodated more balanced sequencing output. Over all our advice is to aim for 3M PE sequence reads for all sample types which results in sufficient meta‐reference clusters, an efficient monoculture‐based cluster filtering, proper calibration and robust estimation of species abundances of the mock‐mixture‐ and unknown experimental samples. Using qPCR based pooling allows for 120–140 samples to be processed in a single Hiseq X‐Ten sequence lane.

#### 4.1.2 Meta‐reference assembly

The number of GBS reference clusters generated depends on the sequencing effort, the restriction enzyme choice, clustering parameters and genome related properties of a species. In msGBS, the restriction enzyme choice cannot be optimized per species. For the Jena field study data we observed a high variation in the number of clusters generated per species ranging from 8,475 for *Geranium pratense* to 260,885 for *Phleum pratense*. However, this was strongly correlated to the sequencing effort. For the Dutch field study, were we aimed for 3M sequence reads per sample, we observed much less between species variation in cluster numbers. Despite the large variation, sufficient clusters were yielded for the Jena field study to allow robust estimation of the across‐ or within‐species abundance. Overall, we do not regard cluster number variation as a fundamental problem since the mock‐mixture reads are all mapped to the same set of clusters.

#### 4.1.3 BLASTN filtering

The processing of the Jena field study meta‐reference BLASTN output led to the removal of only 1% of the clusters. Removed clusters were predominantly annotated to AM Fungi and Bacteria (60% and 34%, respectively). Many clusters could not be identified because of the incompleteness of the NCBI nr database used. A demonstration of this is that >99% of the removed AM Fungi clusters had a hit against *Rhizophagus irregularis* strain DAOM_181602 = DAOM_197198; the only AM Fungi (Tisserant et al., 2013) of which genome‐scale sequence information is present in the NCBI nr database. The monoculture material of the Dutch field study, for meta‐reference assembly, was collected from aboveground leaf material to prevent unnecessary interference with soil biota.

#### 4.1.4 Mapping

We used assembled (merged and joined) reads for mapping instead nonassembled reads. We believe that, for msGBS, assembled reads is preferable; some fragments are in the size region were 20 bp overlap, needed for a read to merge, is just present for some read pairs but not for others resulting in merged and joined variants of the same locus. During clustering those variants are not collapsed and therefore result in more than one cluster. The mapping of assembled reads prevents bias as they will only map to either the merged or nonmerged variant cluster of that locus.

#### 4.1.5 Monoculture‐based cluster filtering

Evaluation of the per cluster read counts in the CSV file showed that it was quite common that monoculture reads of multiple species were mapped to a single meta‐reference cluster. Monoculture‐based cluster filtering identifies clusters with relative high nontarget mapping. Nontarget mapped reads can be caused by (a) between species homologous clusters; (b) clusters that originated from root‐ or rhizosphere microbiota; (c) nontarget roots present in the monoculture plots or laboratory environment pollution; and (d) tag‐ or index jumping (Schnell et al., 2015) although this is mainly a problem in library types that have blunt‐end ligation steps in the wet protocol. Oram et al. (2018) reported that the Jena field study monoculture material of *Holcus lanatus* and *Poa pratensis* contained traces of *Plantago lanceolata*. Due to the monoculture‐based cluster filtering, we found no significant elevated signal for these species using msGBS. The monoculture‐based cluster filtering lowered the FPS. However, a low number of clusters (e.g. in *Geranium pratense)* will cause the monoculture‐based cluster filtering to be less effective; the detection of between species homologous clusters is only possible when those clusters are present. As a consequence mock‐mixture sample *G. pratense* reads which are not represented in the *G. pratense* meta‐reference cluster set, might map to other species clusters causing a higher FPS in those species. This might explain the higher average FPS reported for pool 2.

For the Jena field study, we accepted a maximum of 6.7% (f2 = 15) nontarget reads resulting in high between msGBS and qPCR correlations, acceptable FPS, no FNS and minimal sample loss due to filter f3 (1,000 reads). For the Dutch field study, were we had a higher monoculture sample read average, we accepted a maximum of 0.33% (f2 = 300) nontarget reads resulting in low rFPS and only two discarded samples due to filter f3 (2000 reads).

#### 4.1.6 msGBS in noncalibrated and calibrated mode

Our results showed that msGBS in noncalibrated mode resulted in slightly lower correlations between biomass‐based species proportions and within‐species abundances compared msGBS in calibrated mode. msGBS in both noncalibrated and mode resulted in low mock‐mixture sample FPS. In general the msGBS results of pool 1 were more robust compared to those of pool 2. We believe this was mainly due to a lower on average sequence effort for the pool 2 samples; especially the insufficient sequencing effort of the pool 2 *Geranium pratense* monoculture sample and thus the low number of *Geranium pratense* clusters in the meta‐reference which is hypothesized to result in a less efficient monoculture‐based cluster filtering and higher FPS in the nontarget species.

msGBS in calibrated mode delivered results comparable to the qPCR‐based method of Mommer et al. (2008). The Calibration procedure was able to correct for the 22‐fold differences in, across‐species, read mapping counts in the calibration samples of pool 1. Some variation in per species relative sequence read mapping counts was observed between calibration samples. This variation is probably due to the small amounts of root biomass used per species in these samples (12.5 mg per species; eight species), the manually weighing procedure where differences in root morphology and moistness of the monoculture roots created errors. The use of replicate calibration samples enables the removal of outliers to ensure the calculation of a representable “calibration key”. Overall, the across‐species mapping counts between calibration samples were stable within species pools which was a prerequisite for the msGBS in calibrated mode.

#### 4.1.7 False positive and false negative signals (FPS, FNS)

No FNS was detected within the Jena field study mock‐mixture samples in both msGBS mode. The average FPS of the qPCR and msGBS calibrated data were similar for pool 1. For pool 2 a relative high FPS was recorded for *Plantago lanceolata* and *Holcus lanatus* possibly partly due to the low sequencing effort of the *Geranium pratense* monoculture root sample as discussed above. But this is at least partly contradicted by the fact that there was, for both species, a high variation in FPS between samples which directs more to pollution of monoculture field plots. For the analysis of experimental samples of unknown composition low FPS rates are important. The low FPS rates (<1%) observed for pool 1 are acceptable for analysis of field samples; an analytical detection limit of 1% can be introduced. But, based on the results of the Dutch field study, we believe msGBS can perform even better with a higher sequencing effort and the use of leaf material for meta‐reference assembly. The FPS analysis of the Dutch field study cannot be executed in the way of the Jena field study; the biomass‐based abundances of the root samples are unknown. We used the msGBS signals of congener species to review the taxonomic resolution in terms of relative FPS (rFPS) as discussed below.

#### 4.1.8 msGBS taxonomic resolution

Taxonomic resolution is an unresolved issue in plant taxonomy studies due to high homologies between closely related congener species and is further complicated by a plethora of natural hybrids. The use of longer sequences can solve this issue but current long read sequencers do not deliver premium quality reads nor sufficiently read numbers. When using huge numbers of smaller but high quality Hiseq reads many assembled meta‐reference clusters are highly homologous between species. The abundant presence of Bacterial and Fungi in plant roots further complicate species‐specific quantification.

The effect of monoculture‐based cluster filtering, which identifies between species homologous clusters, on the taxonomic resolution is best evaluated at congener species level. Of all current available techniques that target plant material, msGBS is best compared to RevMet (Peel et al., 2019). To our knowledge, RevMet and msGBS are the only HTS based method that uses nontargeted sequencing data (and not an extracted metabarcoding or mitogenome subset) for the quantification of plant species relative abundances within mixed species samples.

The RevMet mapping data of the two congener pairs (*Papaver* and *Ranunculus*; REF) present in their mock‐mixture data set was used to calculate the rFPS. The rFPS of RevMet and the Dutch field study msGBS data were compared to evaluate the taxonomic resolution of both methods (Table 2). For one of the two congener pairs of RevMet, and for three of the 11 congener groups of msGBS a high rFPS (>3%) was recorded. For two of the three high rFPS msGBS cases, the interference was consistent over samples and within a single pair of species, corresponding with close phylogenetic relatedness. Within the *Ranunculus* congener triplet, visual inspection (Figure S8) of the *Ranunculus* msGBS signals confirmed that the rFPS within *R. repens* was solely caused by *R. bulbosus* sequence reads. No interference between the *R. acris* and both *R. repens* or *R. bulbosus* signals was observed, this corresponded to their phylogenetic relatedness (Baltisberger & Hörandl, 2016). Within the *Rumex* congener triplet; visual inspection (Figure S9) of the *R. thyrsiflorus* and *R. acetose* signal showed interference in both directions. For *R. crispus* no significant rFPS was detected from the other two species, this again corresponds to their phylogenetic relatedness (Schuster et al., 2015). More RevMet congener data is needed to properly evaluate the RevMet taxonomic resolution but in the single case were a direct comparison with msGBS could be made (rFPS between *R. repens* and *R. acris*) the latter showed an improved taxonomic resolution.

#### 4.1.9 Genetic variation and natural hybrids

Genetic variation within species may be another source of error. Theoretically, genetic variation can cause bias through (a) variable PCR efficiencies caused by mutations in primer‐ or restriction enzyme binding sites; or (b) erroneous identification. This might also be the case in msGBS. For msGBS analysis at species level we expect very limited genetic bias because of four reasons; (a) the use of thousands of clusters per species; (b) the read mapping is based on 95% identity; (c) the application of monoculture‐based cluster filtering; and (d) the fact that we use universal primers which, during PCR, anneal to the ligated adapters and not to the genetic variable gDNA sequence itself.

Within congener groups natural hybrids are hypothesized to cause a msGBS signal for both hybrid donor species. This signal can be misinterpreted as FPS when the hybrid is falsely classified as either donor species during field survey. Especially during the collection of monoculture plant material, one must be cautious of hybrids.

### 4.2 msGBS application on multispecies samples

The origin of the monoculture roots, from which the calibration samples were assembled, is important for correct calibration of mock‐mixture‐ and unknown experimental samples. The environment in which the monoculture roots are harvested should be similar to the experimental conditions with regard to soil type, growth conditions and plant age. Species pool slightly affected the relative sequence read mapping counts of individual species. This was demonstrated by comparing the calibration key values of the three species present in both pools to the slope of the regression model of the biomass‐based species proportions and noncalibrated msGBS estimated per species abundance. For optimal calibration it is advisable to produce calibration samples separate for each experimental condition and timepoint (season, year). To minimize the chance that, in a natural field setting, species are missing in the meta‐reference the monoculture material for the creation of the meta‐reference is best collected aboveground and throughout the year. Species with latent presence in the field plot in the form of seeds or tubers will not interfere with the msGBS signals as roots are first washed from the soil core. Significant FPS signals from missing species are only expected when the species are present in the form of roots and when closely related to species in the meta‐reference.

Sampling representative pure species‐specific fine root tissue in high diversity plant communities in natural field settings will often be difficult to impossible. We have shown that even without the preferred calibration, msGBS can provide meaningful results on quantitative distribution differences for the species in the plant community. For example, the within‐species relative to total sequence read mapping counts can be compared between samples of different locations and soil depth. In this way, the distribution of roots of a single species in the soil column can be compared to soil type, soil heterogeneity and the presence of other species. Likewise, the degree of clustering of roots in the horizontal plane may unravel spatial niches belowground that cannot be derived from aboveground patterns because roots generally have a much wider range than shoots. Although root quantities cannot be compared between species, root distributions can, by which positive or negative associations may be unraveled related to questions of species competition and facilitation. These new opportunities for studying belowground community assembly in relation to environmental change now open up even for most diverse plant communities such a species‐rich grasslands (Frank et al., 2010; Kesanakurti et al., 2011) and tropical forests (Jones et al., 2011).

In conclusion, our results highlight msGBS in calibrated mode as a novel, robust and cost‐effective approach to estimate across‐species abundances in mixed root samples. We showed that msGBS can as well be used in noncalibrated mode to estimate within‐species abundances in high diversity plant communities when the arduous assembly of calibration samples is not preferred. msGBS has a high taxonomic resolution and is well able to distinguish congener species. However, the genetic distance between closely related congener species approaches to the within‐species genetic distance and the genetic gap is in some cases filled by a spectrum of hybrid variants. Although msGBS was developed with plant roots in mind the methodology is applicable to other sample types like pollen‐ or diatom mixtures.

## AUTHOR CONTRIBUTIONS

C.W. designed the research, C.W., and A.S performed the laboratory work, T.G. M.P., and C.W. contributed to analytical tools, C.W., and H.K. analysed the data, C.W., H.K., E.V., L.M., and A.W. wrote the manuscript.

## Supporting information

Supplementary Material

## Data Availability

Raw sequence data can be downloaded from NCBI Sequence read Archive (SRA) (BioProject ID PRJNA604964). All scripts used were made available on GitHub (https://github.com/NielsWagemaker/scripts_msGBS/tree/msGBS-1.0). All further important metadata are available via Dryad (https://doi:10.5061/dryad.m63xsj3xz). See Supporting Information file for metadata details.
