# Supplementary material for: msGBS: A new high‐throughput approach to quantify the relative species abundance in root samples of multispecies plant communities
Source: Mol Ecol Resour. 2020 Nov 12;21(4):1021–36. doi: 10.1111/1755-0998.13278 (PMC8246947; doi:10.1111/1755-0998.13278)
Supplement: Supplementary file 1 — Supplementary Material [file MEN-21-1021-s001.docx]

**Supplemental Information for:**

msGBS: A new high-throughput approach to quantify relative species abundance in root samples of multi-species plant communities

Authors

C.A.M. Wagemaker^1^, L. Mommer^2^, E.J.W. Visser^1^, A. Weigelt^3,4^, T.P. van Gurp^5^, M. Postuma^2^, A.E. Smit-Tiekstra^1^, H. de Kroon^1^.

Addresses

^1^ Institute for Water and Wetland Research, Department of Experimental Plant Ecology, Radboud University, Heyendaalseweg 135, 6525 AJ Nijmegen, The Netherlands

^2^ Plant Ecology and Nature Conservation Group, Wageningen University and Research, PObox 47, 6700 AA, Wageningen, The Netherlands

^3^ ﻿Department of Systematic Botany and Functional Biodiversity, Institute of Biology, University of Leipzig, Leipzig, Germany

^4^ ﻿German Centre for Integrative Biodiversity Research (iDiv) Halle-Jena-Leipzig, Leipzig, Germany

^5^ Naktuinbouw, Sotaweg 22, 2371 GD Roelofarendsveen, The Netherlands

***Glossary***

**Clustering** – The grouping of related sequences into a consensus sequence called clusters. A clustering algorithm is used to collapse DNA sequence reads within a certain percentage of similarity (in this supplement referred to as 95% identity) to create a consensus DNA sequence read called a (DNA) cluster.

**Consensus sequence** – A sequence in which SNP bases are represented as the most common one.

**DNA cluster** – a single DNA sequence that is the product the clustering of a collection of DNA sequence reads of high similarity.

**SNP** – Single Nucleotide Polymorphism.

**Trimming** – The removal of unwanted nucleotides from a sequence read. In our case we trimmed remaining adapter traces and low-quality nucleotides.

**Sequence read mapping** – Sequence read mapping is the process of comparing DNA sequence reads to a DNA reference based on a Burrows-Wheeler (Burrows & Wheeler, 1994) transformation aligner (like BWA, Bowtie2 and STAR).

**qPCR** – Quantitative Polymerase Chain Reaction is a common method mostly used to quantify gene expression but also commonly used for quantification of DNA. In general, it measures the amplification of a PCR product through time via a fluorescent signal from which the between samples relative DNA amounts are estimated

**Universal primers** – PCR primers that anneal to between species common DNA regions.

**gDNA** – genomic DNA

**BLASTN search -** Basic Local Alignment Search Tool is a is a program that uses an algorithm to searches a nucleotide database using a query DNA sequence read and identifies local similarity between sequences.

**PCR duplicates** – HTS only sequences a small proportion of the molecules in a (GBS) sequencing library. In msGBS, this sequencing library is the product of a PCR amplification step. When too many amplification cycles are used the HTS sequencing will sequence multiple copies of an original molecule. The library is over-amplified. PCR duplicates should be removed as it can cause a bias in the mapping dataset. Here we used 2x3 random nucleotides, called UMI’s (Unique Molecule Identifier) in the adapter to identify and remove PCR duplicates. In our pipeline this process is done at a per sample and per cluster level.

**Sequencing library** – A collection of DNA fragments sequence ready for HTS sequencing. For msGBS Illumina Hiseq libraries this means gDNA inserts attached to sequence adapters that contain DNA anchors for annealing to the sequencing slide and primer binding sites for sequencing.

***Library preparations and sequencing***

All enzymatic reactions were performed in a PCR machine. During ligation a heated lid was used at lowest temperature available (30 degrees Celsius) and during restriction enzyme reactions a heated lid was used at 37 degrees Celsius. The msGBS libraries are constructed using two indexed adapters suitable for sequencing on a Illumina Hiseq platform (Fig S1 and Table S1). The adapters are designed according to van Gurp et al.(Van Gurp et al., 2016) with some modifications for enzyme choice and the use of 3N wobble nucleotides to identify PCR duplicates. To minimize the possibility of misidentification of samples, as a result of index sequencing- and synthesis errors, all pair-wise combinations of indices differed by a minimum of three mutational steps, index lengths were modulated from 4 to 6 bp to maximize nucleotide the balance of the first ten bases in order to improve the Illumina raw data analysis. As a consequence of using non-phosphorylated adapters, each DNA fragment–adapter connection contained a single stranded nick (Fig S1; yellow dot).

Extended lab protocol:

The protocol was altered in a number of ways of which the restriction enzymes used and the adapter design were the most relevant changes. Per sample, 300 ng gDNA was digested overnight (17hrs) at 37°C in a volume of 40 µL containing 1x FD buffer (B64, Thermo Scientific, The Netherlands), and 1 uL of both *Pac*I (TTAAT^TAA)(R0547L, New England Biolabs (NEB), England) and *Nsi*I (ATGCA^T)(R0127S, NEB). Following digestion, indexed “wobble” adapters were ligated to the fragments. Combining the 18 BA *Pac*I and 14 CO *Nsi*I adapters (Alpha DNA, Canada) resulted in a maximum of 252 index combinations (Fig. S1 and Table S1). The adapters each contain a 3bp random nucleotide region creating a small wobble after the adapter annealing process. For ligation 4 uL of both BA and CO indexed adapters (600 pg/uL), 6 uL T4 DNA ligase buffer, 0.5 uL T4 DNA ligase (M0202M, NEB) and 5.5 uL of distilled water was added to the 40 μL digestion mix. Ligation was performed for 3hrs at 22°C followed by 4°C overnight. All reactions were pooled, mixed and divided in 8 aliquots. The total volume of each pool was reduced to 40 µL using Qiaquick PCR cleanup (28104, Qiagen, The Netherlands) and size selected by a 0.8x Agencourt AMPureXP (A63880, Beckman coulter, Canada) purification using 22 µL lowTE buffer for elution. A nick translation reaction repaired the fragment–adapter nicks and re-assembled the 5-prime attached adapter strand which directly “unwobbles” the adapters using the opposite adapter strand as template (Fig S1). The enzymatic reaction (1h at 15°C) was performed in a volume of 25 µL containing 19.25 µL purified library, 2.5 μL 10 mM dNTP Mix (N0447L, NEB), 2.5 μL NEBuffer 2 and 0.75 μL DNA polymerase I (M0209, NEB). For each aliquot the library amplification was performed in four replicate 10 µL reactions containing 1 µL nick repaired DNA, 5 µL KAPA HiFi HotStart Sequence readyMix (KK2602, Roche Diagnostics, Switzerland) and 3 pmol of each Illumina PE PCR Primer.

Forward primer : 5’-aatgatacggcgaccaccgagatctacactctttccctacacgacgctcttccgatct-3’

Reverse primer : 5’-caagcagaagacggcatacgagatcggtctcggcattcctgctgaaccgctcttccgatct-3’

Temperature cycling consisted of 95°C for 3min followed by 14 cycles of 98°C for 10s, 65°C for 15s and 72°C for 15s with a final extension step at 72°C for 5min. Smaller DNA fragments are more efficiently amplified during PCR resulting in the sequencing of only a small portion of the genome. All replicate PCR products were pooled, concentrated using Qiaquick PCR cleanup, size selected by 0.8x Agencourt AMPureXP and quantified using a Qubit® dsDNA HS Assay Kit (Life technologies, USA). The size distribution and quality of the Libraries was assessed on a High Sensitivity DNA chip on a 2100 Bioanalyzer system (Agilent, USA). A qPCR quantification was performed by KAPA Library Quantification Kit for HTS (KK4844, KAPA Biosystems, USA) on a Biorad (The Netherlands) [CFX96 Touch™ Real-Time PCR Detection System](http://www.bio-rad.com/en-us/product/cfx96-touch-real-time-pcr-detection-system) for optimal sequencing output. The libraries were spiked with 10% PhiX DNA to further increase the complexity of the libraries. 2x150bp Paired-End sequencing was executed by Novogene (Hongkong) on a Illumina (USA) Hiseq X-Ten sequencer.

***qPCR sensu Mommer et al. (2008) and Oram et al. (2018)***

*From Oram et al. (2018):*

In all samples, each species was separately amplified by real-time PCR with species-specific primer pairs (in triplicate). Primer pairs for *A. odoratum*, *F. rubra* and *L. vulgare* were used as described in Mommer et al. (2008). Primer pairs for *C. jacea,* *D. glomerata*, *G. pratense*, *H. lanatus*, *Haemanthus pubescens*, *K. arvensis*, *P. lanceolata*, *P. pratense*, *P. pratensis* and *R. acris* were developed using the same protocol as Mommer et al. (2008).

Real- time PCR reactions were performed with HOT FIREPol Eva Green (Solis BioDyne, Tartu, Estonia) qPCR Mix Plus with an addition of 0.94 μM MgCl2, a primer concentration of 60 nM for *A. odoratum* and *C. jacea* and 120 nM for all other species, and 4 ng genomic DNA for *P. lanceolata* or 1 ng genomic DNA for the other species, in a reaction volume of 20 μl. The qPCR program was as follows: 15 min at 95°C; then 41 cycles of 20 s at 95°C, 30 s at 62°C and 15 s at 72°C; and finally a melting curve analysis of 5 s per cycle, starting at 70°C and ending at 91°C with an increment of 0.5°C per cycle.

***
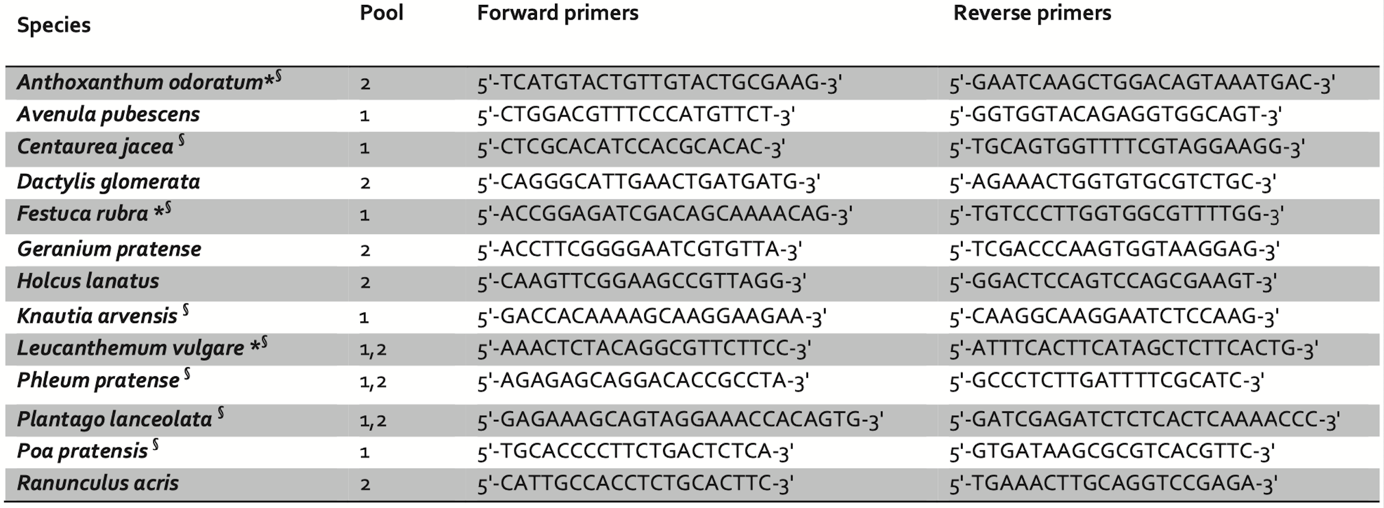
***

qPCR primers used for qPCR based quantification of across-species abundance; Table from Thesis Janneke Maria Ravenek (2015) Belowground species interactions and community effects in species-rich grasslands. PhD thesis, Radboud University Nijmegen, 220p. ^*^ Mommer et al. (2008) and used in Mommer et al. (2010). ^$^ used in chapter 3 and 4 of thesis.

***Extended bioinformatics***

*General information*

Computations were executed on a local cluster node containing two Intel(R) Xeon(R) CPU E5-2450 0 @ 2.20GHz, 512GB RAM and 14 TB hard disk space, using ubuntu 16.04. Editing scripts and debugging was locally performed using PyCharm Professional 2017 2.2 using Python (Python core Team, 2015). R (Suhl et al., 2014) was executed using Rstudio (RStudio Team, 2016).

The msGBS data processing can be partitioned in 5 processes which are outlined in figure 2. Process 1 describe the pre-processing of the raw sequence reads. Process 2 describe the creation and BLASTN filtering of the meta-reference genome from monoculture root material. Process 3 is the mapping of all sequence reads (Fig. 2, product 1) to the filtered meta-reference (Fig. 2, product 2) which result in the BAM alignment file (Fig. 2, product 3). Process 4 is the processing of the UMI’s in the BAM alignment header line, the conversion of the BAM file to CSV format and the monoculture-based cluster filtering which identifies and removes, between species, homologous clusters within the CSV FILE. In the final process 5 the within-species abundance is calculated (Fig. 2, product 5a) and optionally calibrated using the ‘calibration key’ which results in across-species abundance (Fig. 2, product 5b).

The analysis in this article were performed using a series of scripts and commands as described in detail below and are available on [https://github.com/NielsWagemaker/scripts_msGBS branch msGBS-1.0](https://github.com/NielsWagemaker/scripts_msGBS%20branch%20msGBS-1.0). A new, more efficient and easy to install (Anaconda, 2016), snakemake (Köster et al., 2012) version of the pipeline is also made available on GitHub (<https://github.com/NielsWagemaker/scripts_msGBS> using the msGBS-snake branch).

The msGBS-snake Bioinformatics pipeline, essentially doing the same as the msGBS-1.0 scripts, can be installed in four easy steps. Example files and further instructions can be found on <https://github.com/NielsWagemaker/scripts_msGBS> master branch (msGBS-snake branch). This whole msGBS-snake pipeline consists of several independent commands and scripts which are executed using the snakemake (Köster et al., 2012) workflow management system. The combined use of conda and snakemake makes setting up and running the pipeline accessible for beginning Linux users:

- Step one is to clone the msGBS github repository from your linux based terminal.

git clone https://github.com/NielsWagemaker/scripts_msGBS.git

- Step two is to install conda (Anaconda, 2016) on your linux system.
  - See for instructions: <https://docs.conda.io/projects/conda/en/latest/user-guide/install/linux.html>
- Step three is to create a environment in which all dependencies will be installed and from which the pipeline is run and subsequent activation of the environment
  - conda env create -f src/env/environment.yaml --name msGBS
  - conda activate msGBS
- Step four is Run the pipeline
  - Make barcode file
  - Fill in the config.yaml file
  - Run <snakemake -j 12> (and will use 12 cores)

The Analysis steps and scripts of the original msGBS-1.0 pipeline as used for this article are explained in detail below:

***Process 1 : Sequence read pre-processing***

*Demultiplexing*

The first step of the pre-processing (Fig. 2, process 1) was performed by the script Demultiplex_msGBS.py. The input files of the script are the zipped fastq sequence read1 (R1) and sequence read2 (R2) files obtained from Novogene and a tab delimited ‘index or barcode sheet’ that contains sample information (a.k.a. sequence read group information), the indices for the adapters (BA and CO), the number of UMI nucleotides for identification of PCR duplicates (BA and CO) and the used enzymes (BA and CO). An example barcode sheet along with instructions on how to create the correct file type can be found on GitHub. The script removes the index and UMI nucleotides from the R1 and R2 sequence reads and adds them to the sequence information line of each sequence read (BC:Z: and RN:Z: info tags, respectively). The index information is used to extract the sequence read group information from the index file (RG:Z: info tag). The output files of the script are the zipped fastq sequence read1 (R1) and sequence read2 (R2) demultiplexed output files.


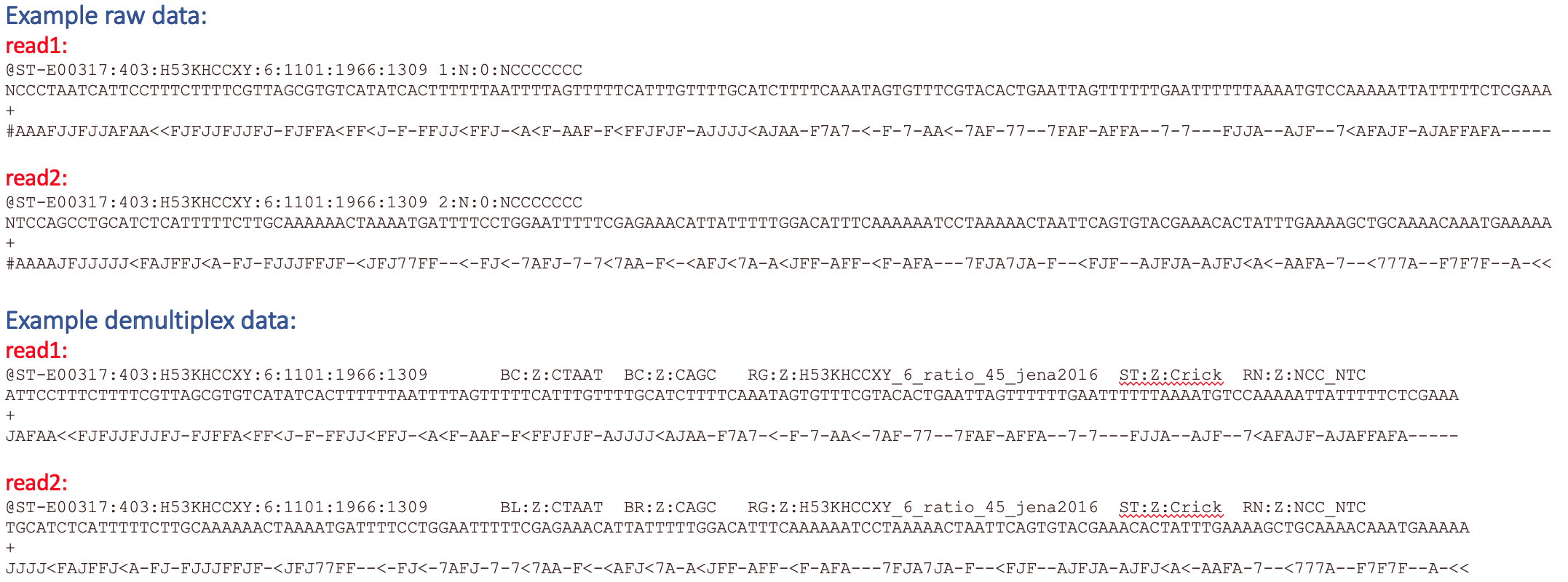
 Not in article referenced figure. The example raw sequence read 1 and 2 show the sequence read information line, the sequence read sequence line, a separation line (+), and the sequence read quality line. The sequence read information line is the combination of the Sequencer code (@ST-E00317:403:), the Flowcell code (H53KHCCXY), lane number (6), sequence read coordinates (1101:1966:1309) and Illumina index information (1:N:0:NCCCCCCC, which we not use as we use ‘inline indices’), respectively. The example demultiplex data shows the additional information tags; BA and CO indices (BC:Z:CTAAT BC:Z:CAGC), sequence read group information (a.k.a. sample name)(RG:Z:H53KHCCXY_6_ratio_45_jena2016), strand identifier (ST:Z:Crick) and wobble nucleotides (RN:Z:NCC_NTC).

*Adapter trimming*

The second step of the pre-processing is the removal of adapter remnant sequences and the trimming of low quality nucleotides (Fig. 2, process 1). The program AdapterRemoval (Schubert, Lindgreen, & Orlando, 2016) was used following the below mentioned parameters. AdapterRemoval --file1 R1_demultiplex_NGmerge_H53KHCCXY_s_6_fastq.txt.gz --file2 R2_demultiplex_NGmerge_H53KHCCXY_s_6_fastq.txt.gz --basename R1_N12_ H53KHCCXY _NG2_no_adapter_ --trimns --trimqualities --minquality 10 --minlength 100 --adapter-list adapters.txt --gzip

***Process 2 : Meta-reference creation and BLASTN filtering***

*Meta-reference creation*

For each monoculture sample in the experiment the python script Make_reference_msGBS.py assembles clusters and creates a reference output file (Fig. 2, process 2). The clusters of all monoculture references are combined into a single meta-reference file while retaining original monoculture identifier names and cluster numbers.

The script first finishes the pre-processing using NGmerge (Gaspar, 2018) in ‘stitch’ mode for merging the R1 and R2 sequence reads using the default parameters (Fig. 2, process 1). In default NGmerge requires that a valid alignment to have a minimum overlap of 20 bp and a maximum of 10% mismatches in the overlap region (-m 20 -p 0.1). NGmerge outputs the merged sequence reads and the R1 and R2 non-merged sequence reads. A log file (in our case a 190 GB file with our 16GB merged.gz file) can optionally be produced for evaluation of all merging events. The quality lines of the reads are retained during this process. In the overlapping stretches the highest quality bases are retained.

The non-merged sequence reads were then joined and a ‘NNNNNNNN’ sequence was inserted between the R1 and R2 sequence reads. The R2 sequence reads were not reverse complemented. The combined set of merged and joined sequence reads of all samples is product 1 of figure 2. For each monoculture sample the merged- and joined sequence reads were extracted from Figure 2, product 1 and separated into single files per monoculture and dereplicated using Vsearch (Rognes, Flouri, Nichols, Quince, & Mahé, 2016) using the following parameters : -derep_fulllength <file_fa.gz_in> --sizeout --minuniquesize 2 -output <output_fa_file>. The resulting merged- and joined files were then combined into a single file per monoculture. These files were then sorted with Vsearch using the following parameters: vsearch --sortbylength <input_fa_file> --output <output_fa_file>. Clustering with 95% identity was performed using vsearch -cluster_smallmem <input_fa_file> -id 0.95 --centroids <output_fa_file> -sizeout --strand both. Because of the low sequencing input for the Jena experiment we kept the minunique size at 2 to prevent even lower number of clusters generated. This resulted in, on average, one cluster per 12 input reads. For the Dutch experiment we used minunique size 3. This resulted in, on average, one cluster per 151 input reads. Whether this dramatic effect in number of clusters generated is mainly caused by the adjustment of this parameter or by the different origin of the monoculture material (root versus leaf, respectively) is unknown.

The resulting file was then transformed by the custom python script rename_fast.py. Finally, the references of all ‘monocultures’ were combined into a single unfiltered meta-reference file named ref.fa. This meta-reference file is indexed using Samtools (Li et al., 2009) by using the following parameters : samtools faidx <input_fa_file> creating a ref.fa.fai index file.

*BLASTN filtering*

The meta-reference filtering step (Fig 2, process 2) is performing by a local BLASTN search using the meta-reference against the NCBI nr database using the following command: blastn -query <path_to>ref.fa -db nt -out <path_to>outputblast_kingdoms.txt -num_alignments 1 -num_thsequence reads 12 -outfmt '6 qseqid sseqid pident evalue bitscore sskingdom sscinames length sstart send '. The BLAST output file contains only the highest hit and some BLAST characteristics; Subject Scientific Kingdom and the Subject Scientific Name.

A custom script named blastN_parse_ref_msGBS.py was used to parse the output file and filter out all clusters that were annotated as Bacteria, Archaea or Viruses. For Eukaryota the genus names in the BLAST output file were used to identify arbuscular mycorrhizal (AM) Fungi (based on an AM Fungi genera list in the script), other Fungi (based on the genera extracted from http://www.mycobank.org/), and plants (based on a genera list extracted from http://www.theplantlist.org). A minimum alignment length of 40 bp and an ‘expect value’ of smaller than 1e-20 of the BLASTN hit was required to accept the annotation. A .txt file was written for each of the above groups containing the clusters that were filtered out the reference. The script also creates text files containing a list of the genus names. Non-filtered identified clusters and clusters that gave no hit to the NR database were retained in the meta-reference (Fig. 2, product 2).

***Process 3 : Mapping***

A custom python script named Map_STAR_msGBS.py was used for mapping of the pre-processed reads (Fig. 2, product 1) to the meta-reference file ref.fa (Fig. 2, product 2). Map_STAR_msGBS.py uses the R script STAR (Zaleski et al., 2012). STAR creates a mapping index genome for merged- and joined sequence reads separately using the following parameters:

STAR --runThsequence readN 6 --runMode genomeGenerate --genomeDir <genome_directory> --genomeFastaFiles <genome_directory/fasta_file> --genomeSAindexNbases <locally calculated> --genomeChrBinbits <locally calculated>.

The sequence read mapping by STAR for joined and merged sequence reads independently is performed using the following parameters:

STAR --runThsequence readN 16 --genomeDir <genome_directory> --sequence readFilesIn <non-assembled R1 sequence reads> or <merged sequence reads> --outSAMattributes NM MD AS --outSAMtype SAM --outFileNamePrefix <prefix of output files> --outFilterMatchNminOverLsequence read 0.95 --clip3pNbases 1 --outSAMorder PairedKeepInputOrder --outFilterMultimapScoreRange 0 --alignEndsType EndToEnd --scoreGapNoncan 0 --scoreGapGCAG 0 --scoreGapATAC 0 --scoreDelOpen 0 --scoreDelBase 0 --scoreInsOpen 0 --scoreInsBase 0 --alignMatesGapMax 20.

Using these parameters allows the multimapping of a read to a maximum of 10 clusters but only when they have identical high alignment scores. These setting allow the monoculture-based cluster filtering to be possible. Otherwise detection of between monoculture homologous clusters would not be possible.

After mapping and file reformatting the Map_STAR_msGBS.py script sorts the SAM file using Sambamba (Tarasov, Vilella, Cuppen, Nijman, & Prins, 2017) and saves the output as BAM file (Fig 2. product 3).

***Process 4 : Post-processing of read mapping data***

*The marking of PCR duplicate sequence reads*

The custom script Mark_PCR_duplicates.py uses the UMI nucleotides in de RN:Z: tag of the demultiplexed sequence read pairs to identify PCR duplicates (Fig 1, step 7). When both R1 and R2 wobble code are identical between individual sequence read-pairs these are considered PCR duplicates and marked as ‘is_duplicate’ in the BAM file. Further when sequence reads AS (Alignment Score) was below 0.8 x cluster length the sequence read was marked as ‘qc_fail’ in BAM file.

*BAM to CSV conversion*

The custom script msGBS_STATS.py parses the BAM file and exports a .CSV output file containing the mapped sequence reads counts per clusters for all samples in the experiment. The script discards sequence reads that are marked ‘qc_fail’ and ‘is_duplicate’ and does not extract clusters to which less then 10 reads over *all* samples mapped.

*Monoculture-based cluster filtering*

The monoculture-based cluster filtering, the final step of Figure 2 process 4, is performed by the custom script Parse_csv.py which also produced the products of process 5. The complete synthax is parse_csv.py -i

/Users/NielsWagemaker/Dropbox/Documents/PROJECTEN/HANS/SGBS_barcoding/article_2018/DATA/mapping_Feb_2019_ref095_map095/out_dedup08_stats_726605_clusters.csv -op

<PATH>\msGBS_run1_ -os f8_f15_1000_pool1_no1_STD.csv -f1 8 -f2 15 -f3 1000 -p 1 -e EXTRA which includes input file, output suffix and prefix, the monoculture-based cluster filtering parameters f1, f2 and f3, the pool for process 5 analysis and -e for optional extra output. The monoculture cluster filtering is independent of species pool as all monocultures are evaluated simultaneously.

***Process 5 : Non-calibrated and calibrated analysis***

After monoculture-based cluster filtering parse_csv.py renames the ‘jenamono1-13’ samples to actual species names. The within-species abundances of the mock mixture root samples are calculated and saved in e10_tussen_sum_REL_p<pool_f1_f2>.csv. The calibration key is calculated from the calibration sample read mapping counts (from filtered CSV file) and subsequently used to do calibrated analysis. The across-species abundances of the mock mixture root samples are saved in <prefix>FINAL<suffix>.

***Figures and Tables***

***Library prep and adapters***


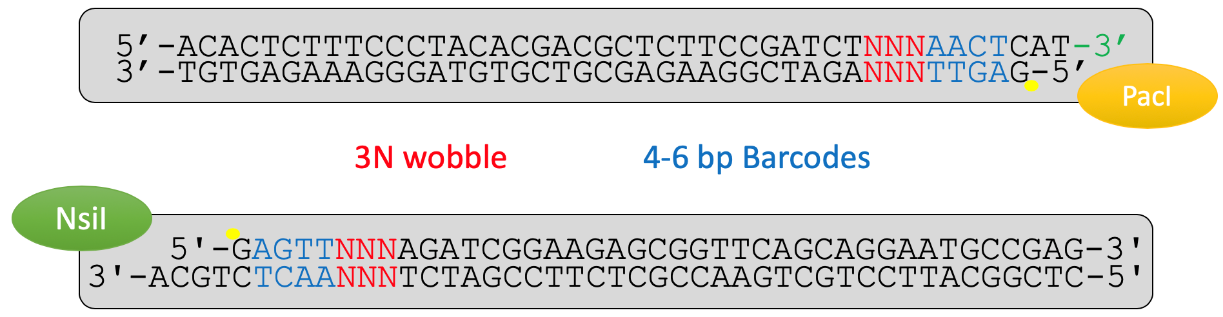


Figure S1. Overview of the BA (upper) and CO (lower) indexing adapters. The blue nucleotides represent the variable index nucleotides used for demultiplexing (Table S1). The red nucleotides represent the random unique molecule identifier (UMI) nucleotides used for PCR duplicate identification. Before nick repair these random nucleotides cause a local mismatch between the adapter strands. The *Pac*I (yellow oval) and *Nsi*I (green oval) represent the complementary overhang of the adapters with the respective restriction enzymes used in msGBS. The yellow dots represent nick’s in the dsDNA before nick repair.


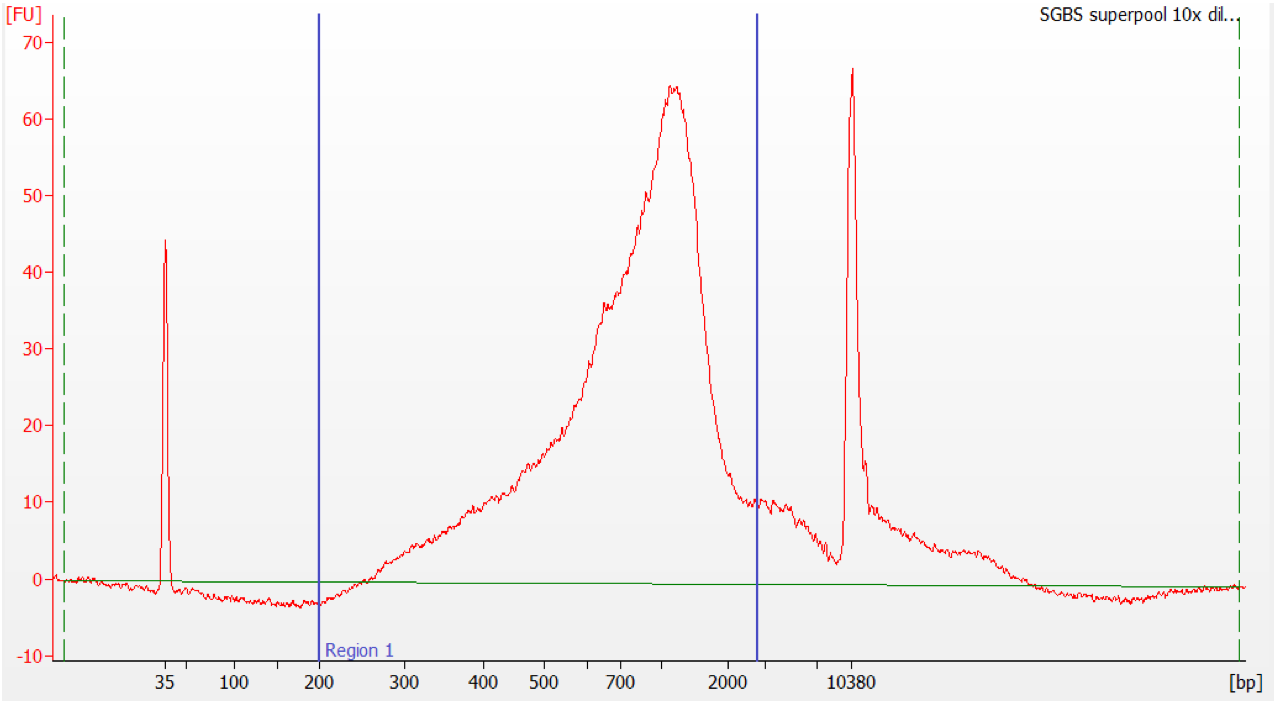


Figure S2. Bioanalyzer result of *the Jena study* msGBS pooled library. Internal Bioanalyzer markers are visible at 35bp and 10,380bp. Smallest library fragments are around 200bp, no adapter dimer was detected (146bp). Above 2,000bp non-amplified fragments are visible. X-axis displays fragment length in bp, y-axis displays fluorescent signal units (FU).

***Meta-reference creation and over-all read mapping statistics***


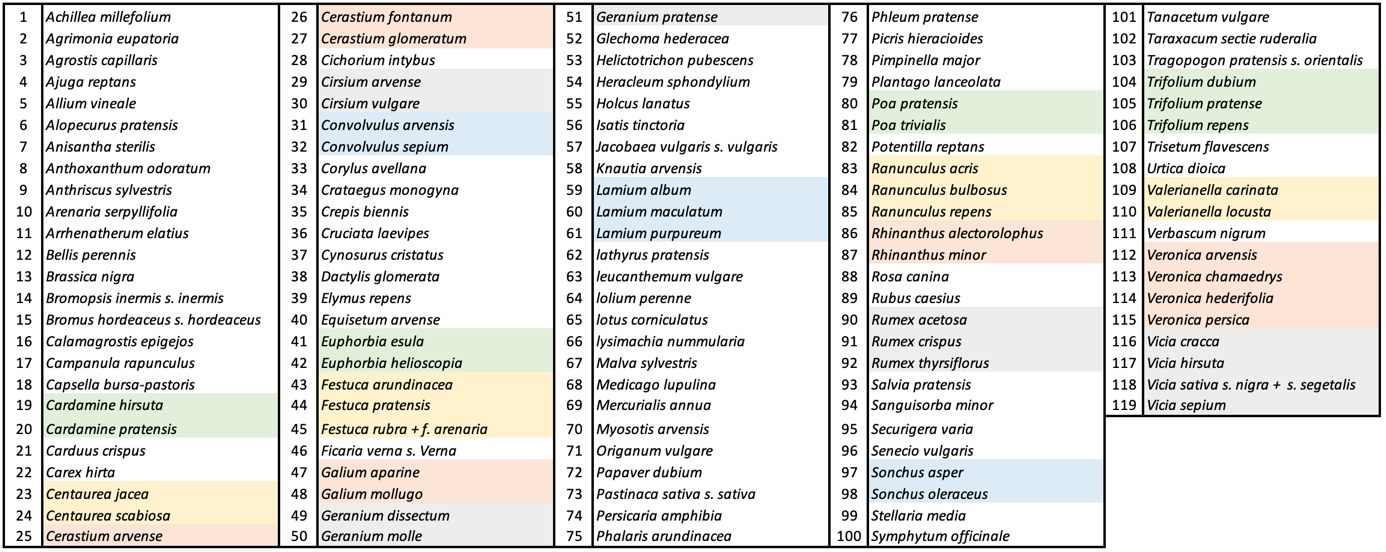
Table S1 Species list of the *Dutch field study* reference species. Coloured are the congener species groups; for 11 congener groups root mixture samples were available for within congener msGBS signal comparison.


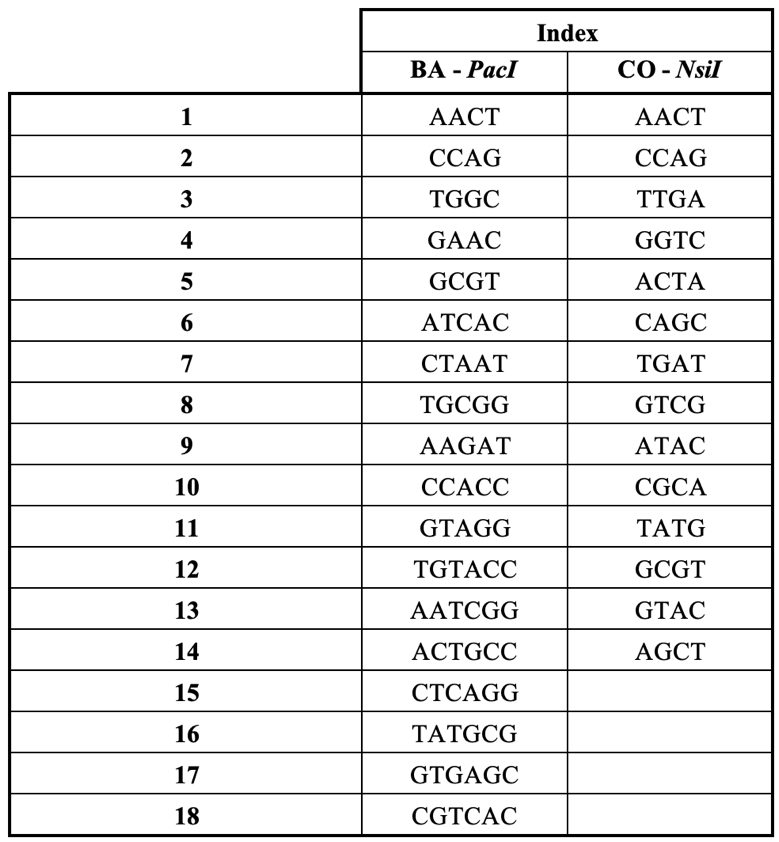


Table S2. Adapter indices of the BA-*Pac*I and CO-*Nsi*I adapters. A maximum number of combinations of 18*14 = 252 can be made. And thus, a maximum of in total 252 samples can be processed in a single Sequencing lane.


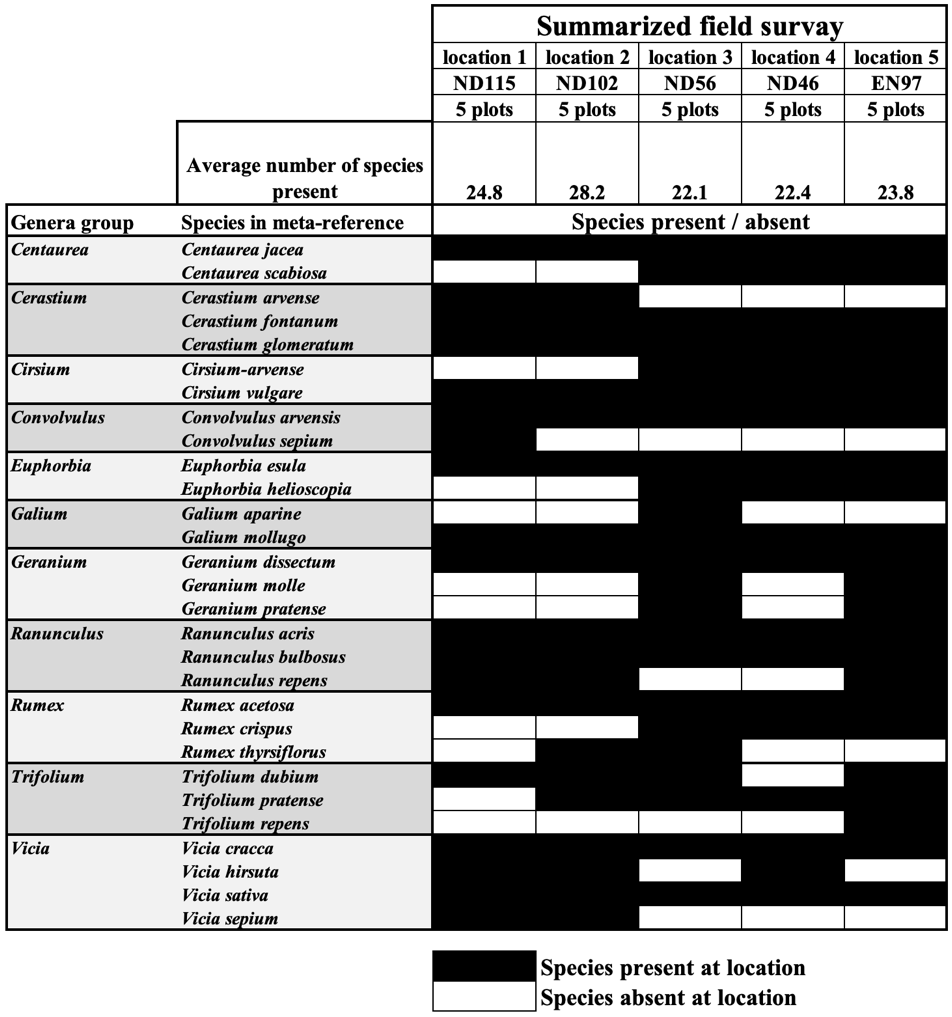


Table S3 The congener species of 11 congener groups and their aboveground presence at location 1 to 5. The total species present in the 5 subplots of each location combined ranged from 22.1 to 24.8 species.


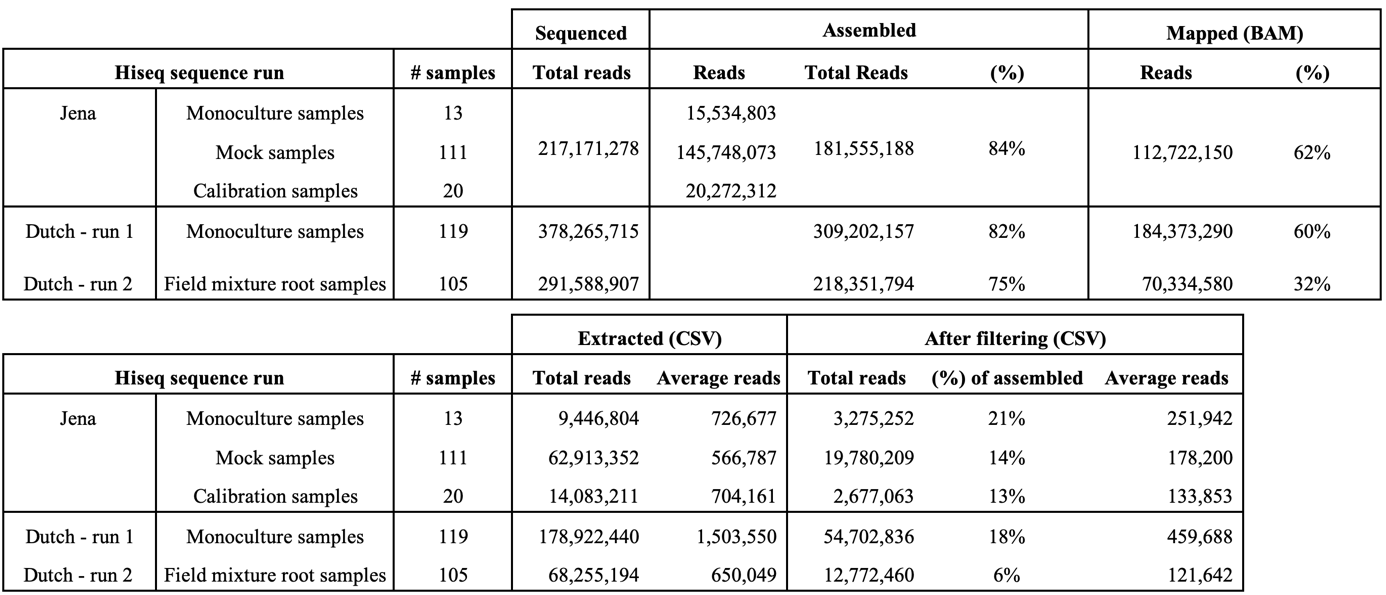


Table S4 Overview of the reads statistics per study and sample type. Displayed are the number of total raw PE sequence reads, assembled reads, mapped reads (in BAM file) and extracted read counts (in CSV file). Mapped sequence reads were not extracted from the BAM file to CSV file when they were annotated as is_duplicate / is_qual_fail or when the cluster to which the sequence reads mapped did not meet the minimum of 10 mapped sequence reads over all samples.


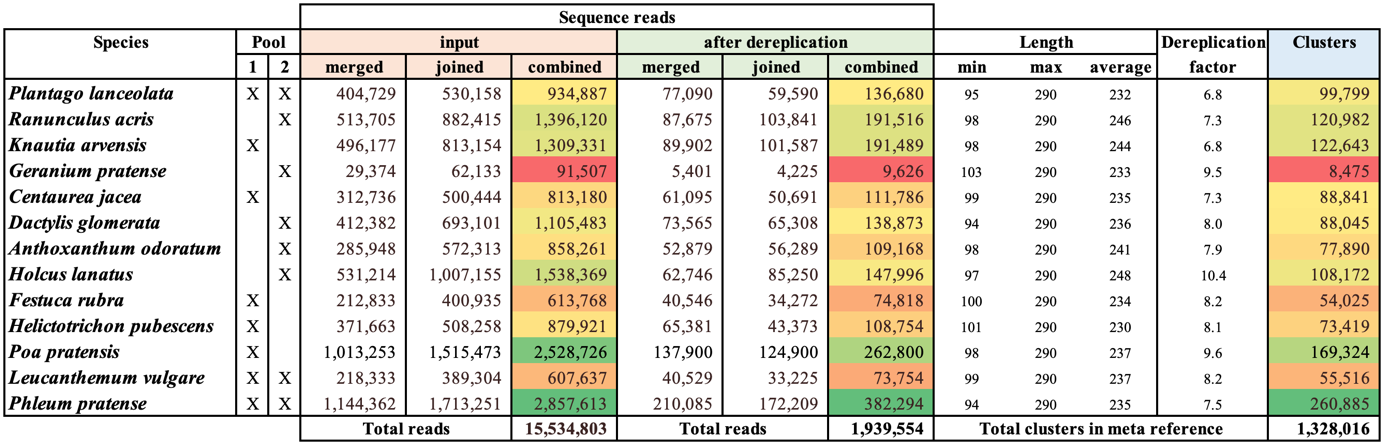


Table S5. *The Jena study* meta-reference input and output reads (merged- and joined) for all species in the meta-reference before and after dereplication. The final column shows the number of Clusters generated after clustering with 95% identity using Vsearch. *Geranium* had low sequence read input which resulted in subsequent low clusters numbers.

***BLASTN filtering***


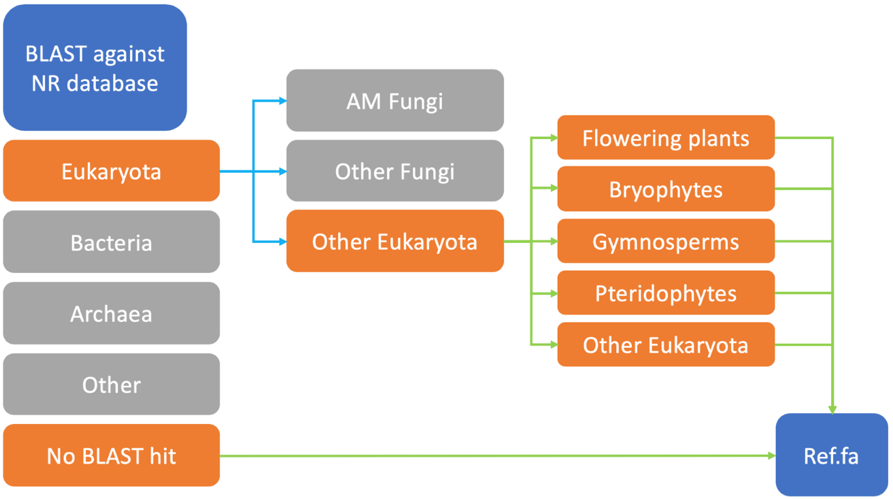


Figure S3. The clusters of the meta-reference were filtered based on the outcome of a BLASTN search against the NCBI NR database. A custom script named blastN_parse_ref_msGBS.py was used to parse ‘Kingdom’ information in the BLASTN output file and filter out all clusters that were annotated as Bacteria, Archaea, Viruses and Phages. For Eukaryota the genus names were used to further identify AM Fungi and other Fungi (genus names retrieved from http://www.mycobank.org/) and plants (genus names retrieved from <http://www.theplantlist.org>). Orange boxes represent clusters that are retained in the metareference, the gray boxes represent clusters that are filtered out.


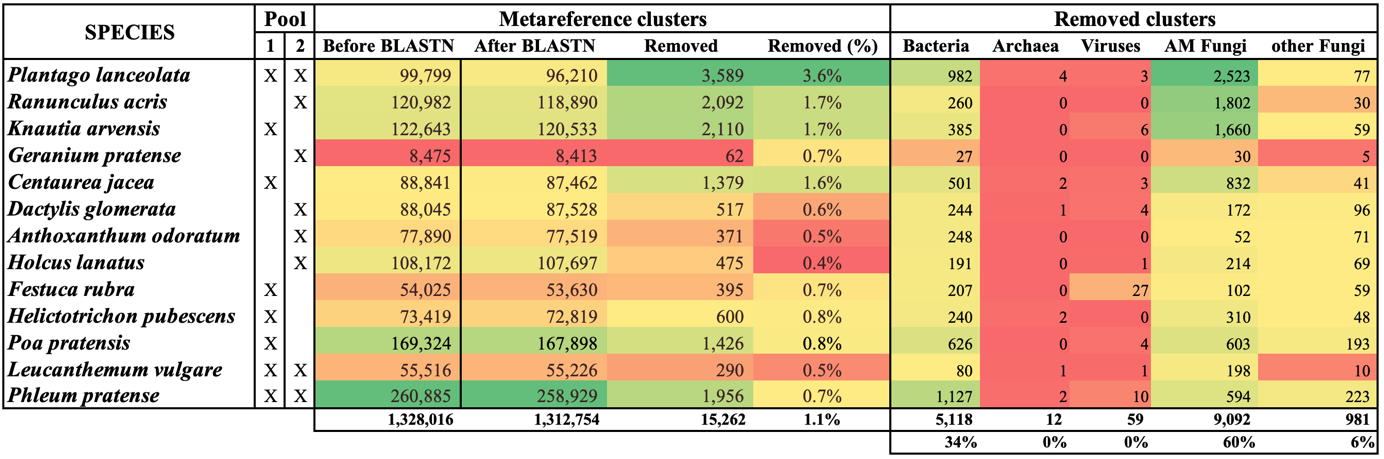


Table S6. Number of *the Jena study* meta-reference clusters before and after BLASTN (against NR database) filtering including the ‘annotation’ of the BLASTN hit per monoculture. Most removed clusters were annotated as AM Fungi with Bacteria as second. Almost all AM Fungi clusters that were removed had hits to *Rhizophagus irregularis* strain DAOM_181602=DAOM_197198 genome.

***Mapping***

***Monoculture-based cluster filtering***

In total the *Monoculture-based* cluster filtering of the *the Jena study* meta-reference removed 513,238 of the 726,605 clusters in the CSV file resulting in the filtered CSV file (Fig 2, product 4). 88,217 clusters were removed due to the prefilter, 389,679 by application of filter parameter f1 and 35,342 after evaluation of filter parameter f2 (Table S6). The filter parameter f2 is only effective for clusters to which enough reads are mapped; we therefor set filter parameter f1 to 8 reads; a balance between effective monoculture-based cluster filtering and the yield of high enough read counts for final quantification. Still, if no non-target reads are recorded it can be that they is not detected due to the low read count. As discussed the low number of *Geranium* reads is expected to result in higher FPS; the *Geranium* root material will contain *Geranium* reads that homologous to other species clusters which were not identified due to the low monoculture read numbers.


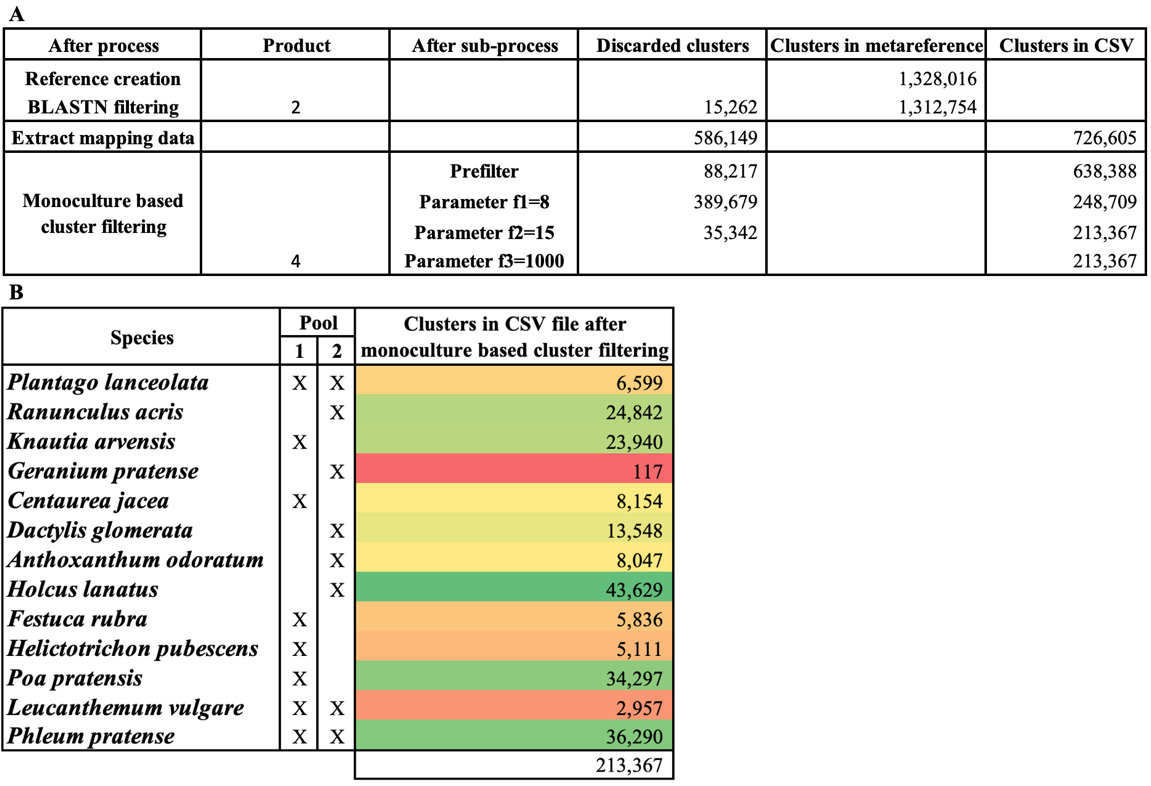


Table S7 Number of clusters of *the* *Jena study* in the meta-reference before and after BLASTN filtering and in the extracted CSV file before and after monoculture-based cluster filtering (A). The number of clusters in the meta-reference after monoculture-based cluster filtering, split per monoculture.


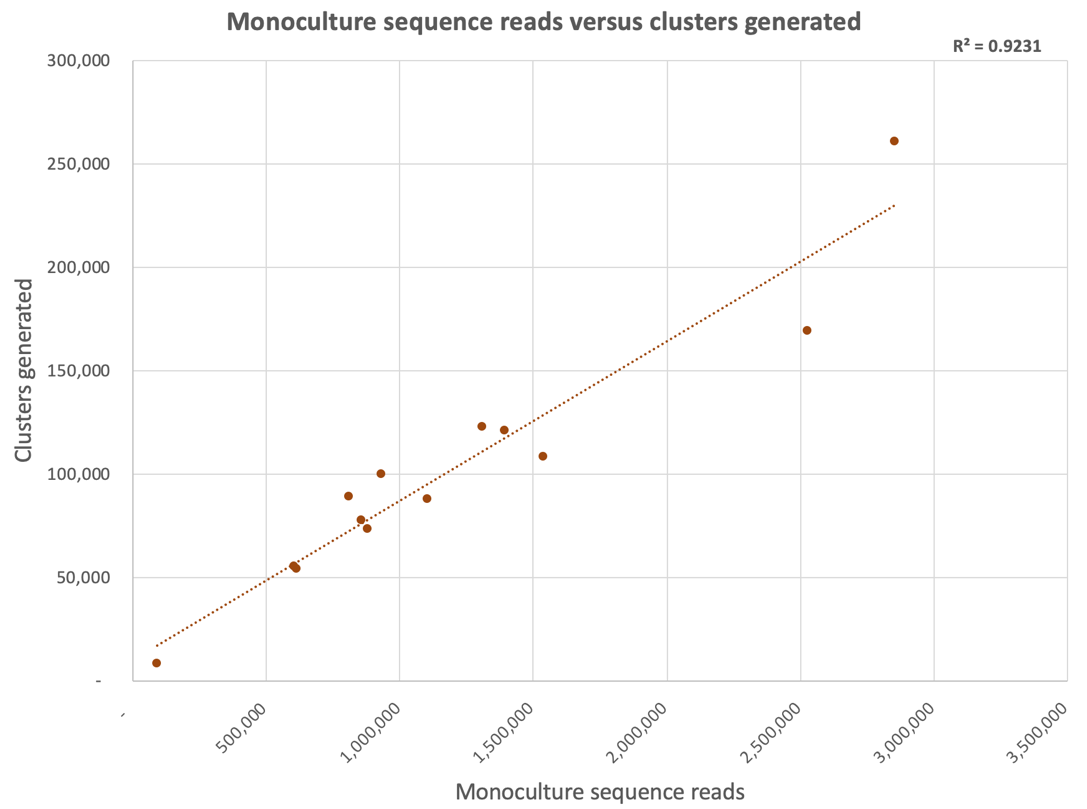


Figure S4 Correlation (R2=0.92) between number of input reads and the number of clusters generated (before filtering) for all 13 monocultures of the *Jena field study*. For most species more sequencing effort will result in more clusters.

***Additional read mapping results***

For the *Jena field study* mapping failure of 27.8% and 17.99% of the merged- and joined sequence reads, respectively, was classified as ‘alignment too short’. Mapping failure of 38.19% and 38.44% of the merged- and joined sequence reads, respectively, was classified as ‘other’ which happens if STAR was not able to find good anchor seeds or if sequence reads mapped to repeat regions. STAR cannot find good anchor seeds if a sequence read is not represented in the meta-reference, for example when a cluster was removed during the BLASTN filtering. This suggests that although not a lot of clusters were removed by BLASTN filtering they represented a high number of reads.

***Non calibrated analysis***


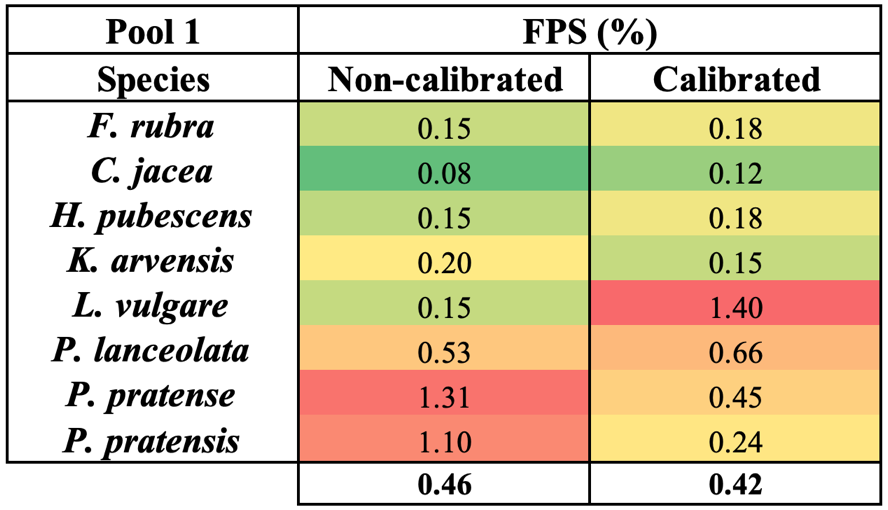


Table S8 msGBS mock mixture sample FPS(%) of pool 1; the average percentage of root biomass estimated in non-calibrated and calibrated mode when species were not supposed to be present (actual monoculture biomass was zero).

***Calculation of the calibration key***


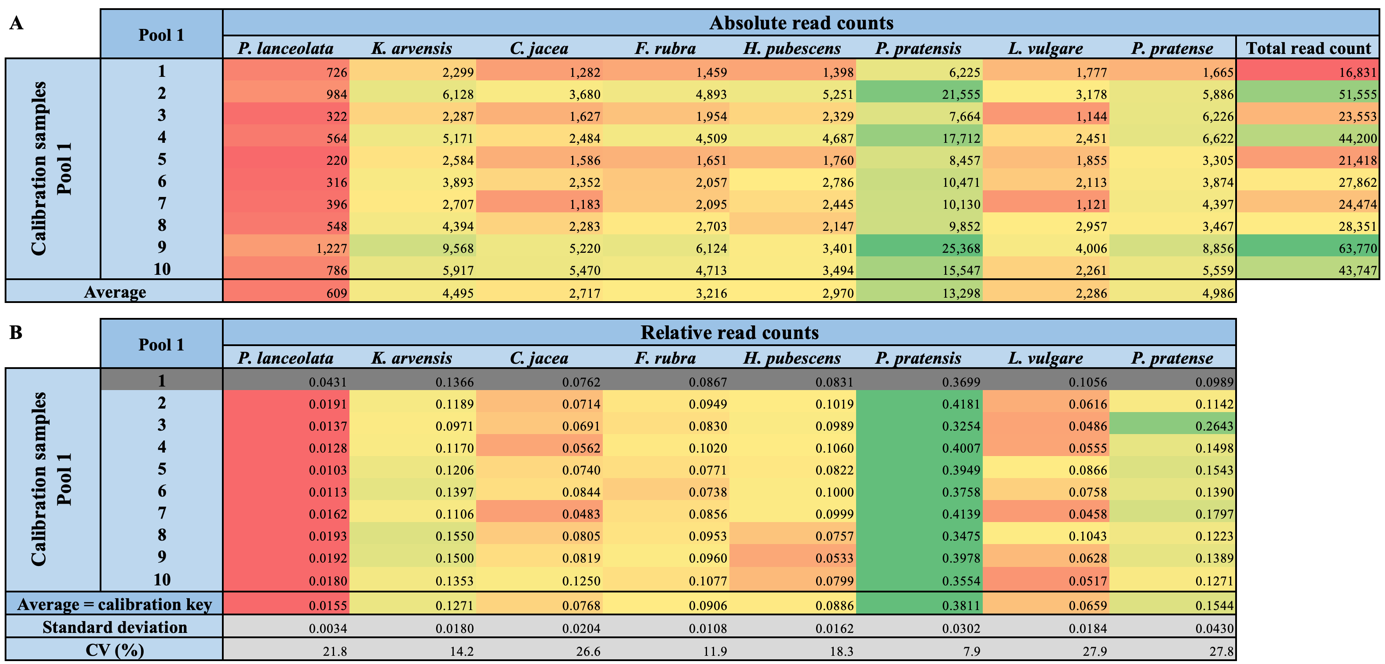


Table S9 The absolute and relative to total read counts of the calibration samples of pool 1. The absolute read counts (A) vary a lot across species. The relative to total read counts (B) of the calibration samples which, if averaged result in the ‘calibration key’. The relative to total read counts vary between species but follow the same pattern across calibration samples. The coefficient of variation (CV), after excluding outlier sample 1 (of which, 1 or more per species values, deviated more than >2.5STD), varies between 7.9 – 27.9%.


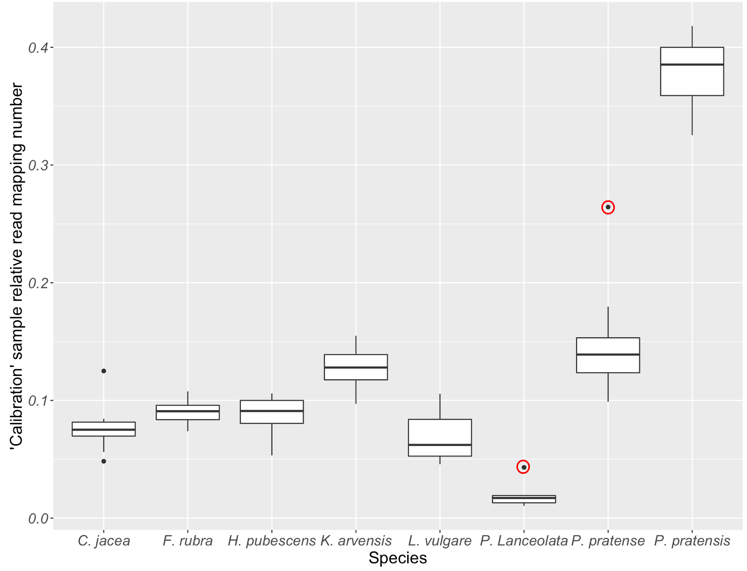


Figure S5 Standard boxplot of the relative mapped read counts of the calibration samples of pool 1 showing the outlier values (>2.5STD) of calibration sample 1 within a red circle.


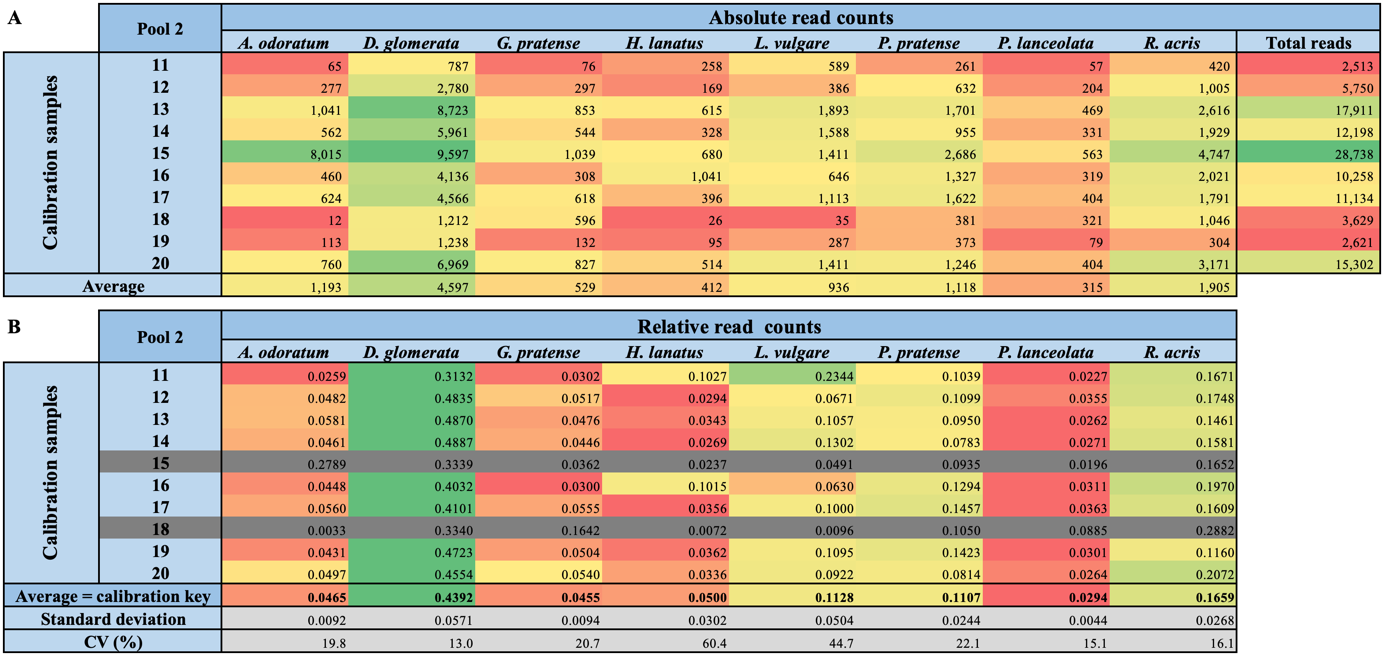


Table S10 The absolute and relative to total read counts of the calibration samples of pool 2. The absolute read counts (A) vary a lot across species. The relative to total read counts (B) of the calibration samples which, if averaged result in the ‘calibration key’. The relative to total read counts vary between species but follow the same pattern across calibration samples. The coefficient of variation (CV), after excluding outlier sample 15 and 18 (of which, 1 or more per species values, deviated more than >2.5STD), varies between 13.0 – 60.4%.


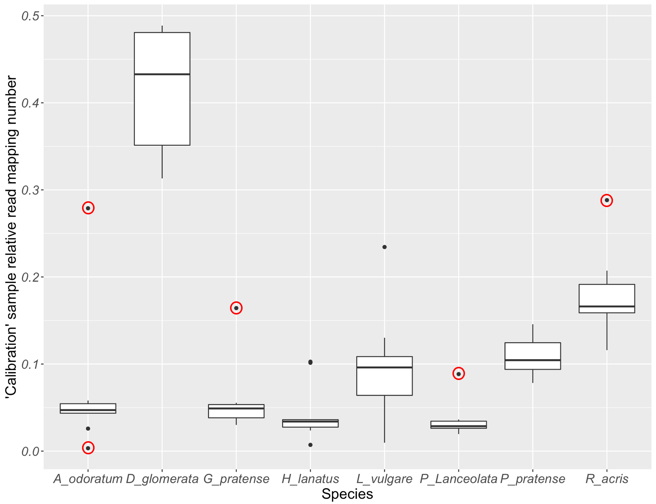


Figure S6 Standard boxplot of the relative mapped sequence read counts of the calibration samples of pool 2 showing the outlier values (>2.5STD) of calibration samples 15 and 18 within a red circle.

***Final analysis of the Jena field study***


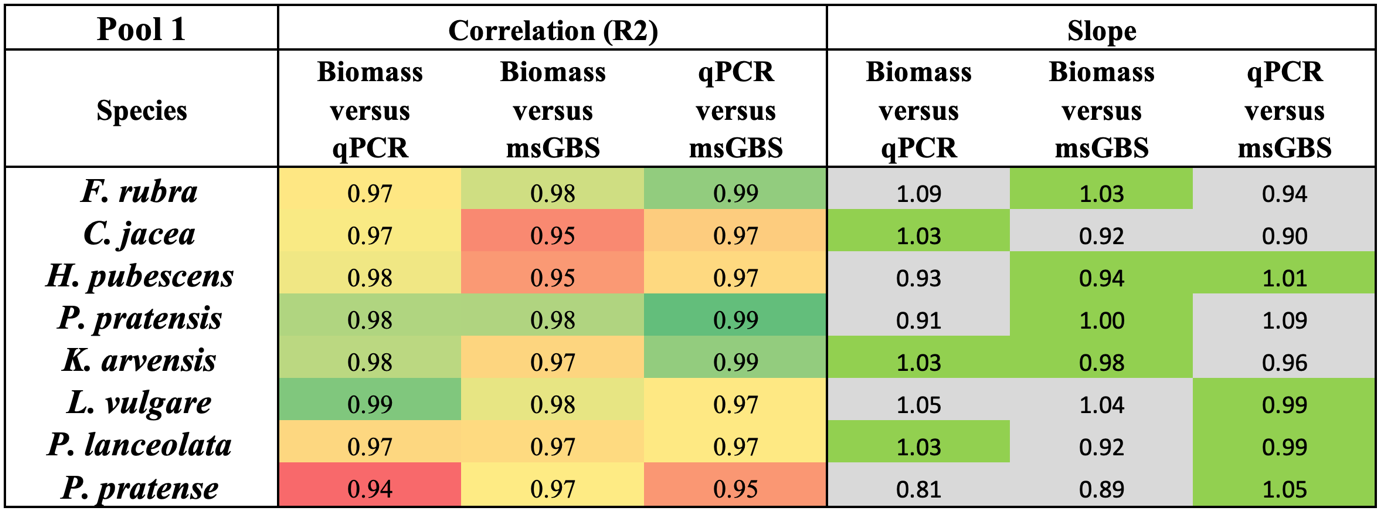


Table S11 Correlations (R2) of root biomass versus qPCR, root biomass versus msGBS and qPCR vs msGBS, respectively, of mock mixture root samples of pool 1. For slopes depicted in green, the slope=1 line was within the 0.95 confidence interval boundaries.


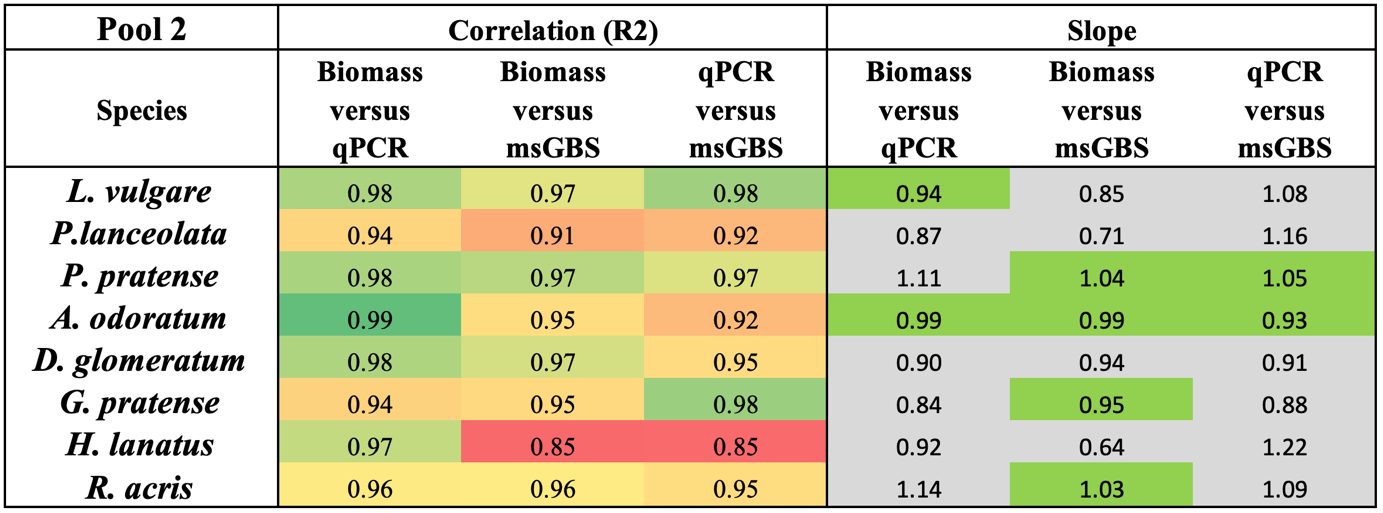


Table S12 Correlations (R2) of root biomass versus qPCR, root biomass versus msGBS and qPCR vs msGBS, respectively, of mock mixture root samples of pool 2. For slopes depicted in green, the slope=1 line was within the 0.95 confidence interval boundaries.


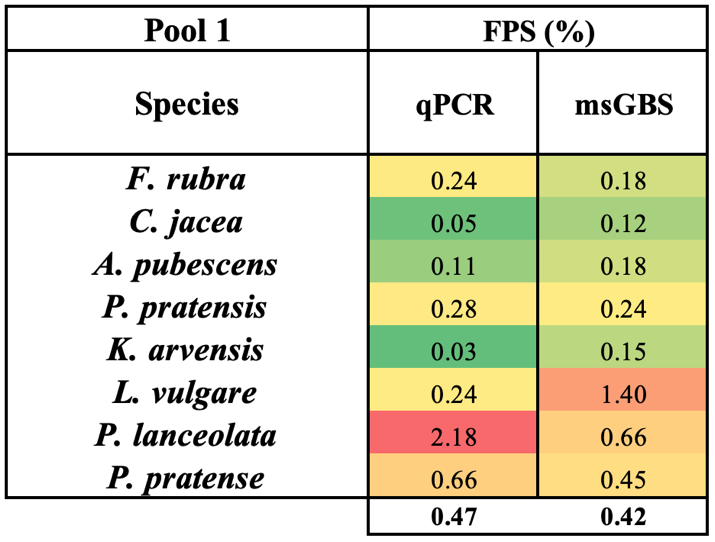


Table S13 Mock mixture sample FPS(%) for pool 1; the average percentage of root biomass estimated when the species was supposed not to be present (actual monoculture biomass was zero), by qPCR and msGBS.


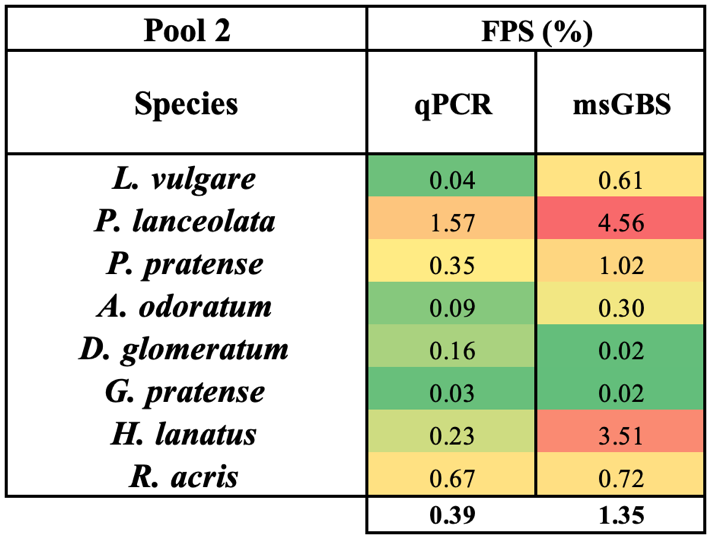


Table S14 Mock mixture sample FPS(%) for pool 2; the average percentage of root biomass estimated when the species was supposed not to be present (actual monoculture biomass was zero), by qPCR and msGBS.


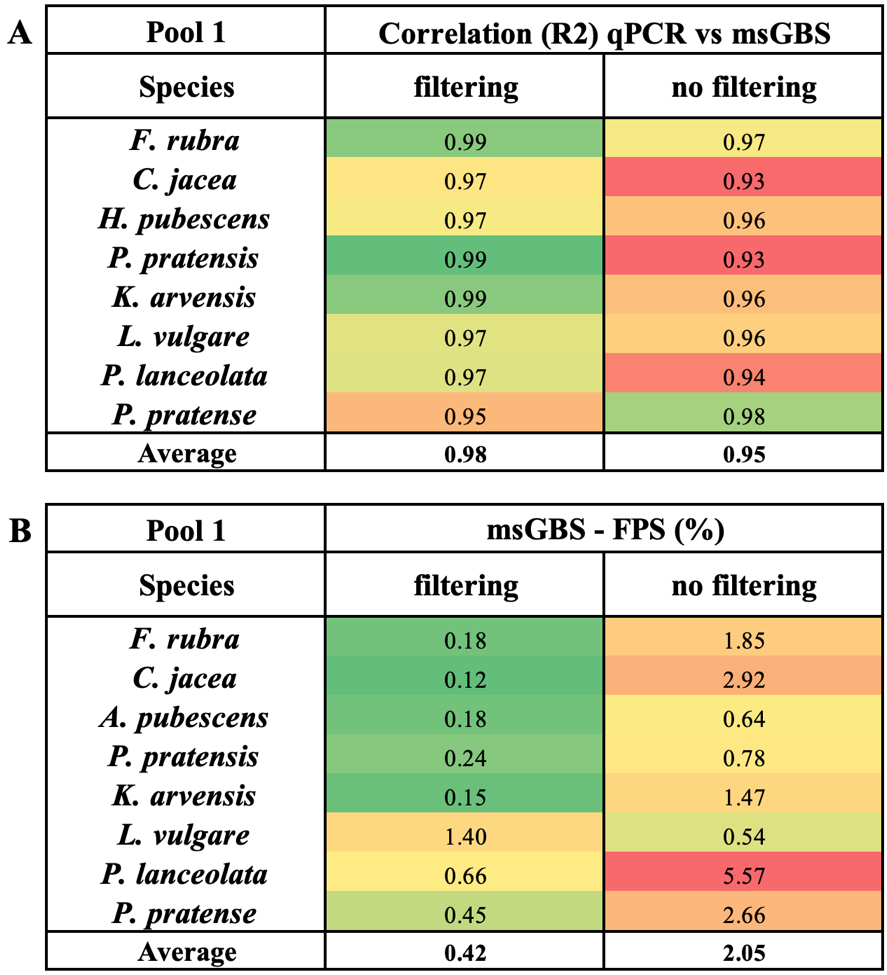


Table S15 The combined effect of the BLASTN- and monoculture mapping based cluster filtering on (A) the correlation of qPCR versus msGBS and (B) the msGBS FPS(%) within species pool 1.


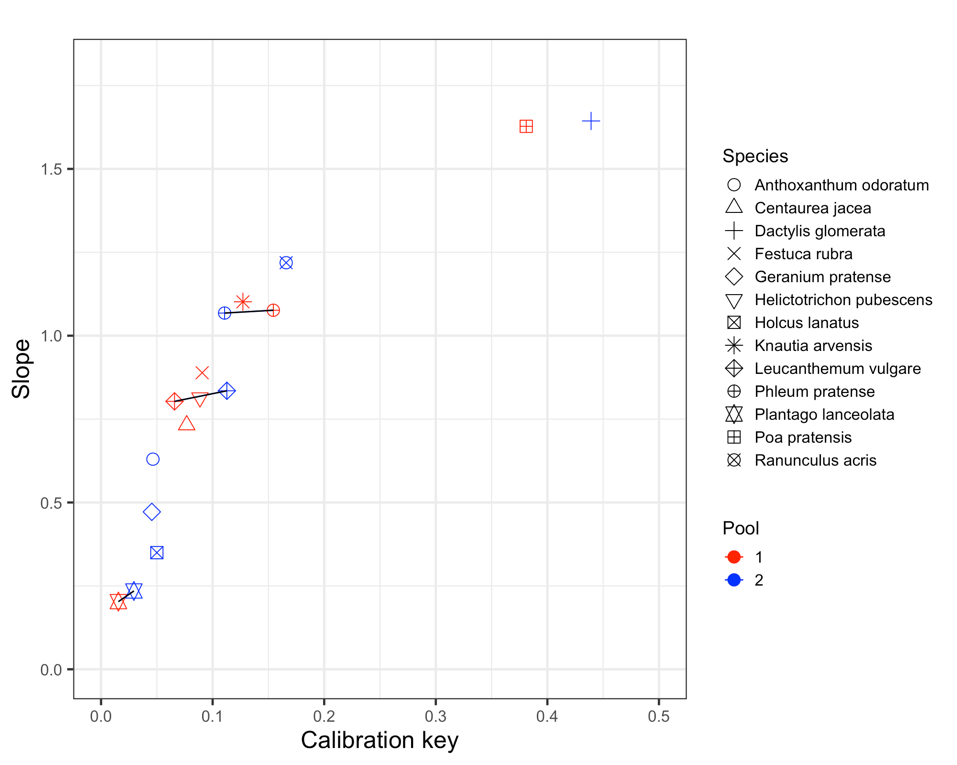


Figure S7 The influence of the species assembly on the calibration key values (Table S5B and S6B) and the non-calibrated regression model slopes (Fig 4) of pool 1 (red) and pool 2 (blue). Symbols of species present in both pools (*Plantago*, *Leucanthemum* and *Phleum*) are connected by black lines. The non-calibrated regression model slopes (of the biomass-based species proportions and non-calibrated msGBS estimated within-species abundance) of the three species present in both pools is hardly altered by the species assembly. For *Leucanthemum vulgare* and *Phleum pratense* the calibration key is shifted, possibly due to the different species assembly.


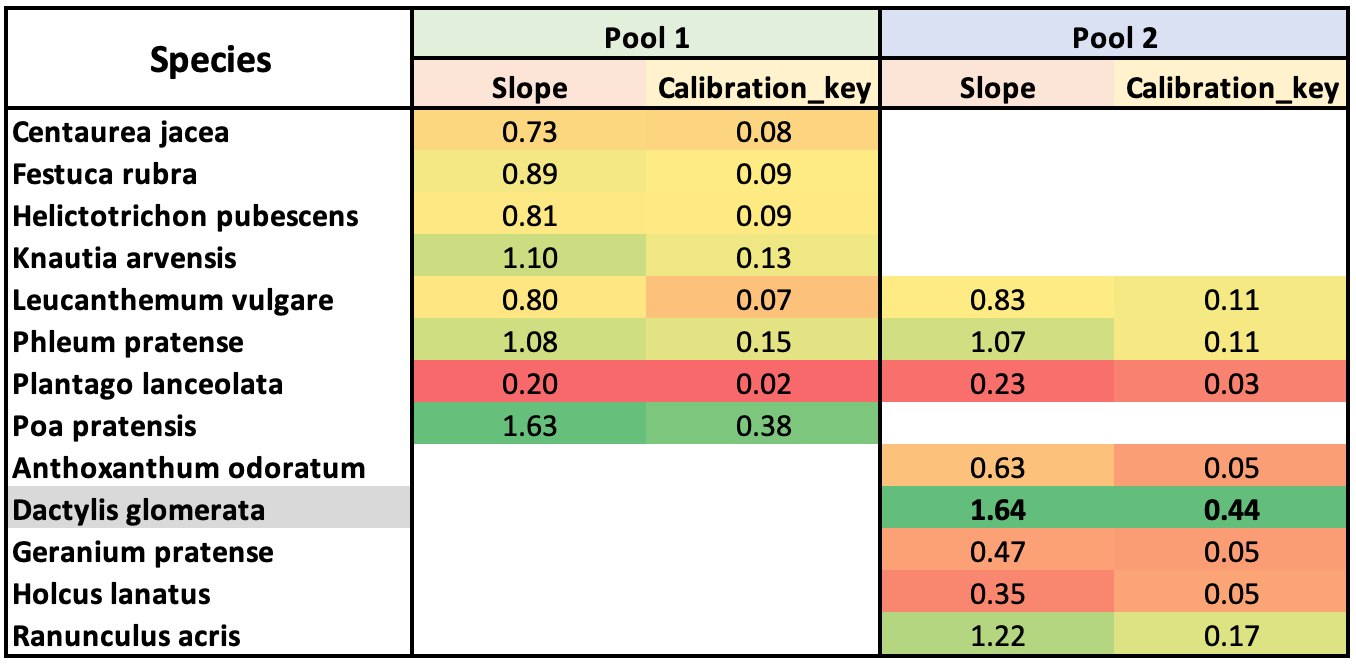


Table S16 The influence of the species assembly on the calibration key values (Table S5B and S6B) and the non-calibrated regression model slopes (Fig 4) of pool 1 and pool 2. The slope and calibration keys of species present in both pools (*Plantago*, *Leucanthemum* and *Phleum*) were compared. The per species shift of the slope ranged from 0.01 to 0.03. The per species shift of the calibration key ranged from 0.01 to 0.4. The slope is less influenced by species assembly compared to the calibration key. However, in relation to other species, the calibration key shift is limited.

***The Dutch Field Study***

To illustrate the data behind the rFPS analysis within congener species groups we included Figure S8 and S9. All monocultures of the *Dutch field study* were represented by a robust set of clusters in the meta-reference. msGBS signals of species not expected to be present within a sample (based on extensive field surveys) are assumed to derive from congener species. This assumption is expected to cause a comparable rFPS between samples. However this was not always observed (for examples as discussed for *Cirsium*). Also in the Ranunculus example below the rFPS is not always present. It can be discussed if the rFPS might also not be caused by hybrid species. However for the closely related congener pair *Ranunculus bulbosus* and *Ranunculus repens* (Figure S8) no hybrids are reported in literature (Coles, 1973). For the closely related congener pair *Rumex acetose* and *Rumex thysiflorus* (Figure S9) hybrids are reported in literature (Świetlińska, 2015).


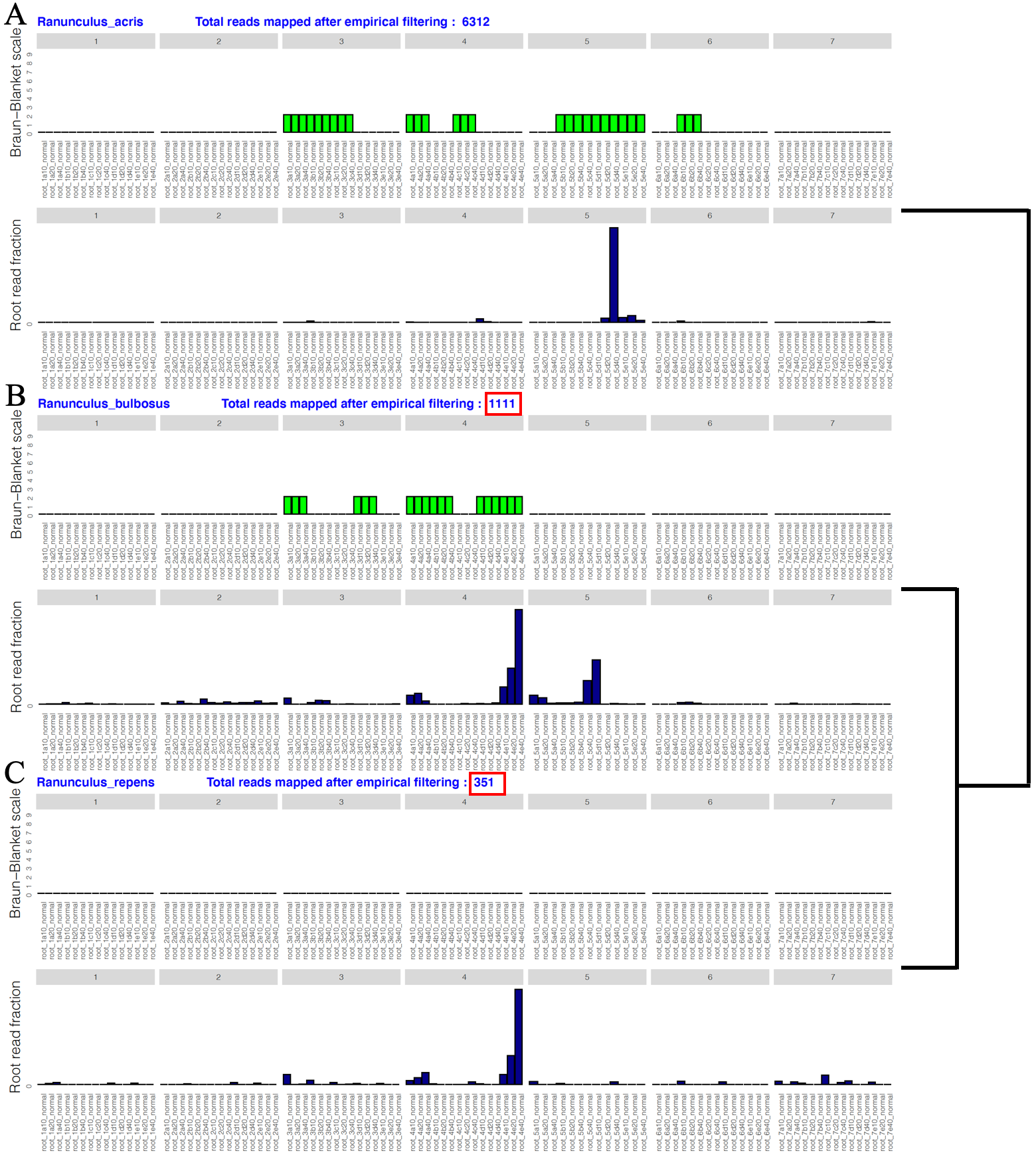


Figure S8 Congener analysis. Example of the *Ranunculus spp.* Field survey of plot 1-7 (ABC-upper; in numeric 0-9 Braun-Blanquet scale) and msGBS (ABC-lower) within species abundance of 7 field locations, 5 plots per location and 3 soil core depths per plot. The phylogenetic relatedness is plotted to the right of the graphs. The *Ranunculus acris* msGBS signal leaves no FPS imprint in the *Ranunculus bulbosus* and *Ranunculus repens* msGBS signal, this correspond to their phylogenetic relatedness (Schuster et al., 2015). It looks as if *Ranunculus bulbosus* derived reads leaves an imprint (44% on average) in the *Ranunculus repens* msGBS analysis, this correspond to their phylogenetic relatedness. However, the *Ranunculus bulbosus* msGBS signal of location 5 subplot c and d did not leave an imprint in *Ranunculus repens* and did also not correspond to the field survey data. It is of course a possibility that a seedling, a seed or an underground root organ is missed during the field survey.


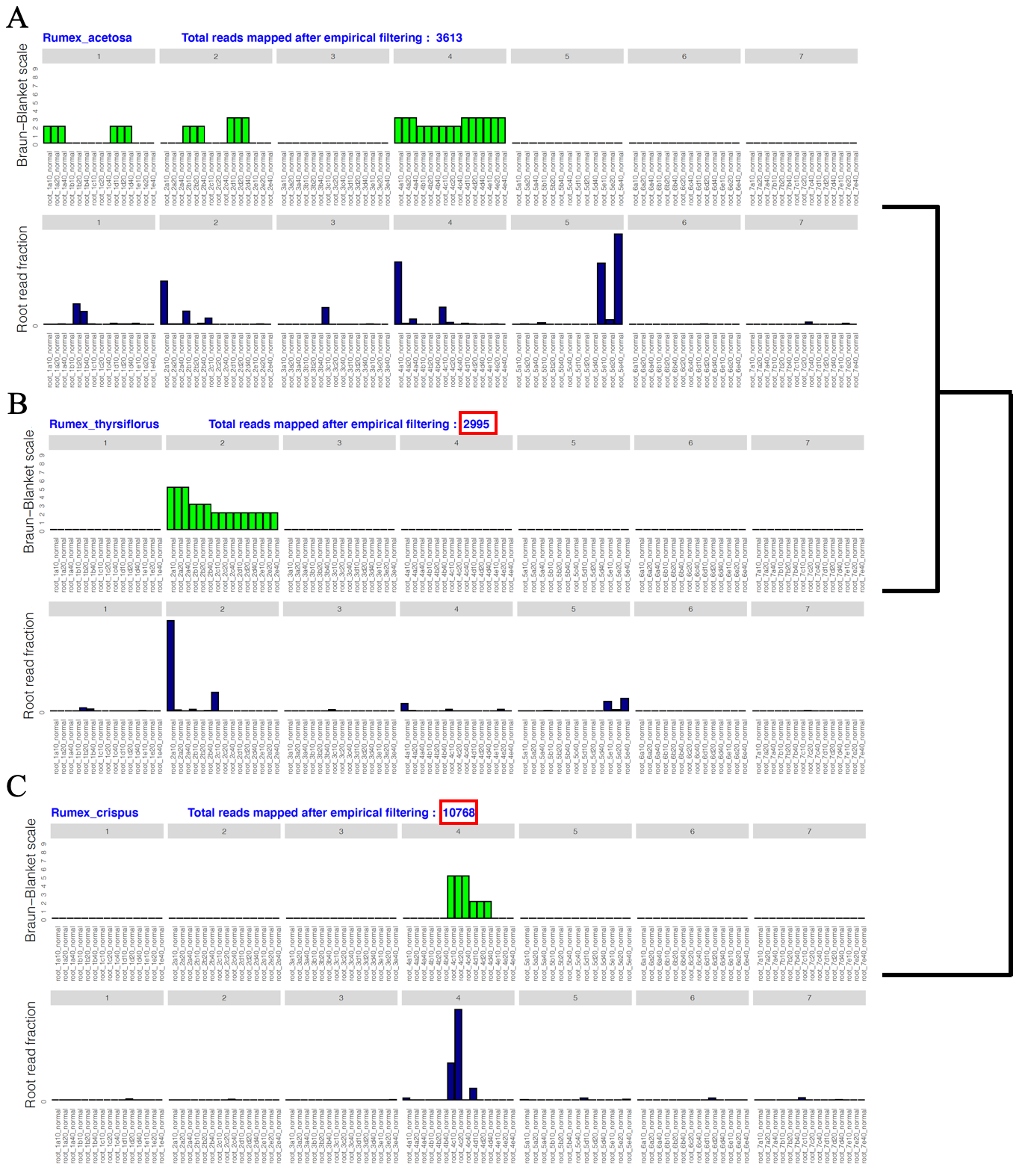


Figure S9 Congener analysis. Example of the *Rumex spp.* Field survey of plot 1-7 (ABC-upper; in numeric 0-9 Braun-Blanquet scale) and msGBS (ABC-lower) within species abundance of 7 field locations, 5 plots per location and 3 soil core depths per plot. The phylogenetic relatedness is plotted to the right of the graphs. The *Rumex acetosa* and *Rumex thyriflorus* msGBS signals leave no FPS imprint in the *Rumex crispus* msGBS signal, this correspond to their phylogenetic relatedness (Baltisberger & Hörandl, 2016). It looks as if *Rumex acetosa* derived reads leaves an imprint (31% on average) in the *Rumex thyriflorus* msGBS analysis, this correspond to their phylogenetic relatedness. However, for example, the *Rumex acetosa* msGBS signal of location 5 subplot e did not correspond to the field survey data. It is of course a possibility that a seedling, a seed or an underground root organ is missed during the field survey.

***References***

Anaconda. (2016). *Anaconda*. Retrieved from https://anaconda.com

Baltisberger, M., & Hörandl, E. (2016). Karyotype evolution supports the molecular phylogeny in the genus Ranunculus (Ranunculaceae). *Perspectives in Plant Ecology, Evolution and Systematics*, *18*, 1–14. https://doi.org/10.1016/j.ppees.2015.11.001

Burrows, M., & Wheeler, D. J. (1994). A Block-sorting Lossless Data Compression Algortihm. *SRC Research Report*.

Coles, S. M. (1973). Ranunculus bulbosus L . in Europe. *Watsonia*, *9*, 207–228.

Gaspar, J. M. (2018). NGmerge: Merging paired-end reads via novel empirically-derived models of sequencing errors. *BMC Bioinformatics*, *19*(1), 1–9. https://doi.org/10.1186/s12859-018-2579-2

Köster, J., & Rahmann, S. (2012). Snakemake-a scalable bioinformatics workflow engine. *Bioinformatics*, *28*(19), 2520–2522. https://doi.org/10.1093/bioinformatics/bts480

Li, H., Handsaker, B., Wysoker, A., Fennell, T., Ruan, J., Homer, N., … Durbin, R. (2009). The Sequence Alignment/Map format and SAMtools. *Bioinformatics*, *25*(16), 2078–2079. https://doi.org/10.1093/bioinformatics/btp352

Mommer, L., Wagemaker, C. A. M., De Kroon, H., & Ouborg, N. J. (2008). Unravelling below-ground plant distributions: A real-time polymerase chain reaction method for quantifying species proportions in mixed root samples. *Molecular Ecology Resources*, *8*(5), 947–953. https://doi.org/10.1111/j.1755-0998.2008.02130.x

Oram, N. J., Ravenek, J. M., Barry, K. E., Weigelt, A., Chen, H., Gessler, A., … Mommer, L. (2018). Below-ground complementarity effects in a grassland biodiversity experiment are related to deep-rooting species. *Journal of Ecology*, *106*(1), 265–277. https://doi.org/10.1111/1365-2745.12877

Rognes, T., Flouri, T., Nichols, B., Quince, C., & Mahé, F. (2016). VSEARCH: a versatile open source tool for metagenomics. *PeerJ*, *4*, e2584. https://doi.org/10.7717/peerj.2584

Schubert, M., Lindgreen, S., & Orlando, L. (2016). AdapterRemoval v2: Rapid adapter trimming, identification, and read merging. *BMC Research Notes*, *9*(1), 1–7. https://doi.org/10.1186/s13104-016-1900-2

Schuster, T. M., Reveal, J. L., Bayly, M. J., & Kron, K. A. (2015). An updated molecular phylogeny of Polygonoideae (Polygonaceae): Relationships of Oxygonum, Pteroxygonum, and Rumex, and a new circumscription of Koenigia. *Taxon*, *64*(6), 1188–1208. https://doi.org/10.12705/646.5

Suhl, J. A., Chopra, P., Anderson, B. R., Bassell, G. J., & Warren, S. T. (2014). R: A Language and Environment for Statistical Computing. *Nature*, Vol. 485, pp. 237–241. https://doi.org/10.1093/hmg/ddu272

Świetlińska, Z. (2015). Cytogenetic relationships among Rumex acetosa, Rumex arifolius and Rumex thyrsiflorus. *Acta Societatis Botanicorum Poloniae*, *32*(2), 215–279. https://doi.org/10.5586/asbp.1963.012

Tarasov, A., Vilella, A. J., Cuppen, E., Nijman, I. J., & Prins, P. (2017). Genome analysis Sambamba : fast processing of NGS alignment formats. *Bioinformatics*, *31*(November), 2032–2034. https://doi.org/10.5281/zenodo.13200.Contact

Team), (Python core. (2015). *Python: A dynamic, open source programming language.* Python Software Foundation.

Team), (RStudio. (2016). *RStudio: Integrated Development Environment for R*. Retrieved from http://www.rstudio.com/

Van Gurp, T. P., Wagemaker, N. C. A. M., Wouters, B., Vergeer, P., Ouborg, J. N. J., & Verhoeven, K. J. F. (2016). EpiGBS: Reference-free reduced representation bisulfite sequencing. *Nature Methods*, *13*(4), 322–324. https://doi.org/10.1038/nmeth.3763

Zaleski, C., Jha, S., Gingeras, T. R., Batut, P., Davis, C. A., Chaisson, M., … Drenkow, J. (2012). STAR: ultrafast universal RNA-seq aligner. *Bioinformatics*, *29*(1), 15–21. https://doi.org/10.1093/bioinformatics/bts635
